# Genomic Evolution and Phylodynamics of the Species *Orthomarburgvirus marburgense* (Marburg and Ravn Viruses) to Understand Viral Adaptation and Marburg Virus Disease’s Transmission Dynamics

**DOI:** 10.3390/pathogens13121107

**Published:** 2024-12-14

**Authors:** Claude Mambo Muvunyi, Nouh Saad Mohamed, Emmanuel Edwar Siddig, Ayman Ahmed

**Affiliations:** 1 Rwanda Biomedical Center (RBC), Kigali 11KG St 644, Rwanda; claude.muvunyi@rbc.gov.rw; 2 Pan-Africa One Health Institute (PAOHI), Kigali 11KG St 203, Rwanda; nouh_saad@outlook.com; 3 Unit of Applied Medical Sciences, Faculty of Medical Laboratory Sciences, University of Khartoum, Khartoum 11111, Sudan; emanwelleds389@gmail.com

**Keywords:** hemorrhagic fever, Global Health Security, pandemic, transdisciplinary One Health, genomics, *Orthomarburgvirus* genus, phylodynamics

## Abstract

In this review, we investigated the genetic diversity and evolutionary dynamics of the *Orthomarburgvirus marburgense* species that includes both Marburg virus (MARV) and Ravn virus (RAVV). Using sequence data from natural reservoir hosts and human cases reported during outbreaks, we conducted comprehensive analyses to explore the genetic variability, constructing haplotype networks at both the genome and gene levels to elucidate the viral dynamics and evolutionary pathways. Our results revealed distinct evolutionary trajectories for MARV and RAVV, with MARV exhibiting higher adaptability across different ecological regions. MARV showed substantial genetic diversity and evidence of varied evolutionary pressures, suggesting an ability to adapt to diverse environments. In contrast, RAVV demonstrated limited genetic diversity, with no detected recombination events, suggesting evolutionary stability. These differences indicate that, while MARV continues to diversify and adapt across regions, RAVV may be constrained in its evolutionary potential, possibly reflecting differing roles within the viral ecology of the *Orthomarburgvirus marburgense* species. Our analysis explains the evolutionary mechanisms of these viruses, highlighting that MARV is going through evolutionary adaptation for human-to-human transmission, alarmingly underscoring the global concern about MARV causing the next pandemic. However, further transdisciplinary One Health research is warranted to answer some remaining questions including the host range and genetic susceptibility of domestic and wildlife species as well as the role of the biodiversity network in the disease’s ecological dynamics.

## 1. Introduction

Marburg virus disease (MVD) is a severe hemorrhagic fever with high fatality rates caused by either Marburg virus (MARV) and/or Ravn virus (RAVV), both belonging to the genus *Orthomarburgvirus* of the family Filoviridae [1,2]. Since MARV’s discovery in 1967 during an MVD outbreak in Germany, it has emerged as a major public health concern due to its ability to cause large outbreaks with significant mortality and a high case fatality rate, ranging from 24% to over 88%, seemingly depending on the population or environmental condition per the geographical areas, particularly in many African countries including Angola (2004–2005) [3], the Democratic Republic of Congo (1998–2000) [4], Uganda [5,6], Kenya [7], South Africa [8], Ghana [9], Guinea [10], Tanzania [11], and, most recently, Rwanda for the first time in 2024 [12]. The transmission of MARV and RAVV in human populations primarily occurs through direct contact with body fluids of infected individuals or contaminated objects, and sporadic spillover events from its natural reservoir, the Egyptian fruit bat (*Rousettus aegyptiacus*) [13,14,15].

RAVV was first identified in 1996 and it was considered as a new subtype of MARV due to the similarity in disease progression; however, genomic studies revealed that RAVV is a separate virus from MARV and differed from it by up to 21.3% of its genomic structure; however, both viruses share the same ancestral origin [3,16,17]. MARV and RAVV, like other filoviruses, have a single-stranded, negative-sense RNA genome that encodes seven structural proteins, including nucleoprotein (NP), viral proteins (VP35, VP40, VP30, VP24), glycoprotein (GP), and the RNA-dependent RNA polymerase (L) [3,16,18].

MVD caused due to infection with MARV or RAVV cannot be differentiated clinically. A laboratory diagnosis of MVD includes molecular methods such as the reverse-transcription polymerase chain reaction (RT-PCR), which detects MARV or RAVV viral RNA, or serological tests like the enzyme-linked immunosorbent assay (ELISA) [19]. While RT-PCR provides definitive evidence of active infection, antibody testing can offer insights into past exposures and immunity in the population. However, cross-reactivity with other flaviviruses can complicate serological diagnosis, since other pathogens during outbreaks cannot be easily differentiated unless relying on genomic sequencing tools to differentiate the causative virus [20,21].

To understand MARV and RAVV’s evolution and phylodynamics, viral genomic sequencing plays a pivotal role in identifying mutations and determining transmission dynamics and adaptation during outbreaks. This study aims to investigate MARV and RAVV’s evolutionary history and phylodynamics for understanding how those viruses adapt and spread within human populations and across different regions, as well as identifying patterns of viral evolution and any adaptations that may enhance their fitness or transmissibility.

## 2. Materials and Methods

### 2.1. Data Collection of Viral Genomic Sequences

We collected publicly available genomic sequences of MARV and RAVV isolates of confirmed cases or reservoir hosts during outbreaks from the National Centre for Biotechnology Information (NCBI) GenBank database (https://www.ncbi.nlm.nih.gov/datasets/genome/, accessed on 23 November 2024). Each sequence was accompanied by metadata, including the year of isolation, geographic location, and the host species.

### 2.2. Sequences Selection

To ensure the integrity and reliability of our analysis, we carefully curated the sequence dataset to exclude any isolates that are not complete or partial genomes and/or derived from laboratory-adapted strains or recombinant forms of MARV or RAVV (Supplementary File S1: Table S1). Laboratory-adapted strains often accumulate mutations or genetic changes that arise under artificial conditions, such as repeated passaging in cell cultures or animal models. These mutations can differ significantly from those occurring during natural infection and transmission cycles, potentially introducing biases in evolutionary or genetic diversity analyses [22].

Similarly, recombinant sequences—where genetic material from different viral strains or species has been artificially combined—do not represent naturally occurring genetic exchanges and could obscure patterns of natural evolution. These were also excluded to maintain the focus on authentic evolutionary processes [23].

In addition to laboratory and recombinant exclusions, we ensured that all retained sequences originated from natural reservoirs, such as bats, or from direct human infections documented in outbreaks. This approach was crucial for capturing the genetic variation that occurs during natural spillover events and human-to-human transmission. By doing so, we aimed to avoid confounding factors introduced by unnatural environments or conditions that do not reflect real-world transmission dynamics.

This rigorous selection process aimed to enhance the robustness of the analysis, allowing us to investigate the evolutionary dynamics of MARV and RAVV with greater confidence. It ensures that the observed genetic diversity and evolutionary patterns genuinely represent the natural processes shaping these viruses in their reservoirs and human hosts. As a result, our findings are more relevant to understanding the adaptation, spread, and potential emergence of these viruses in natural contexts, contributing valuable insights for public health strategies and outbreak management.

The final dataset consisted of complete or nearly complete genomes representing multiple outbreaks across various geographic regions and time points. The accession numbers of the sequences analyzed in this study along with their metadata are available in Supplementary File S1: Table S2.

### 2.3. Sequence Alignment and Genetic Diversity Indices

All MARV and RAVV sequences were aligned using MAFFT v7 with default parameters to ensure optimal nucleotide alignment. The alignment was performed using the FFT-NS-2 algorithm, a progressive method optimized for general-purpose alignments and medium-sized datasets, with the number of refinement iterations set to 0. For nucleotide sequences, a substitution matrix with equal weights for matches and mismatches was applied, while default gap penalties were used, with a higher penalty assigned for opening gaps and a lower penalty for extending them. Moreover, manual curation was performed to remove poorly aligned regions and gaps, especially in non-coding or repetitive regions, that could introduce noise into the analysis using Unipro UGENE software (version 51.0) [24]. Nucleotides and the deduced amino acid sequences of the protein-coding regions from MARV and RAVV genomes were analyzed to assess genetic diversity parameters, including nucleotide diversity (π), the number of haplotypes (H), haplotype diversity (Hd), and neutrality test indices such as Tajima’s D [25], Fu and Li’s D, and Fu and Li’s F statistics [26]. We performed sliding window analyses for each virus genus separately, including MARV and RAVV, to investigate patterns of genetic diversity, selection pressure, and evolutionary dynamics across different regions of the genome. For MARV, the analysis was conducted on a subset of 70 sequences, while, for RAVV, 12 sequences were included. The analyses were performed for whole genome sequences as well as their corresponding protein-coding regions using a window size of 25 base pairs with a step size of 10 base pairs to identify specific genomic hotspots of high variability or selection. All sliding window analyses and genetic diversity parameters were calculated using DnaSP software (version 5.10.1) [27].

### 2.4. Phylogenetic Analysis

For phylogeographic analysis, each viral sequence was linked to its corresponding geographic location and the year of isolation. This information allowed the tracing of the virus’s movement over time and space. Geographic data were used to categorize viral isolates into discrete countries, while temporal data were used to calibrate evolutionary models. To assess the evolutionary relationships among MARV and RAVV isolates, a maximum likelihood phylogenetic tree was constructed using MEGA7 software (version 7.0.26) [28], using the Maximum Likelihood method based on the Tamura 3-parameter model with Gamma-distributed rate variation among sites [29]. Bootstrap analysis with 1000 replicates was conducted to evaluate the statistical support of the branching clusters. Sequences used as outgroup taxa when constructing the phylogenetic tree are shown in Supplementary File S1: Table S3.

### 2.5. Selection Pressure and Adaptive Evolution Analysis

To detect evidence of positive or purifying selection acting on the MARV and RAVV genomes, we calculated the ratio of non-synonymous (dN) to synonymous (dS) substitutions (dN/dS) using DnaSP software to detect codon positions subject to differential selection pressures between hosts.

The minimum number of recombination events (Rms) for each gene was also calculated by analyzing linkage disequilibrium and examining patterns of genetic variation across the MARV and RAVV sequences using the Hudson and Kaplan method [30], by estimating the minimum number of recombination events required to explain observed haplotype structures in the data using DnaSP software.

### 2.6. Haplotype Network Construction

To visualize the microevolution of MARV and RAVV within and between outbreaks, we constructed haplotype networks using PopART software (version 1.7) [31] for the identification of mutational relationships between viral isolates and viral diversity and transmission chains based on host, temporal, and geographical representations. The years of outbreak or field investigations for virus isolation were grouped to minimize redundancy and prevent misinterpretation of results. Older sequences were consolidated into a 1967–1987 group, capturing initial outbreak data, while more recent outbreaks were organized into defined intervals: 1998–2000, 2004–2005, 2007–2012, 2013–2018, and 2021–2022. This grouping strategy allowed for clearer temporal analysis and comparison across outbreak periods. Field investigation isolates were incorporated within these groups.

## 3. Results

### 3.1. Neutrality Testing and Genetic Diversity

The neutrality testing of MARV and RAVV populations based on protein-coding regions across different countries and hosts revealed varying results. The MARV population from the DRC which related to humans exhibited a high haplotype diversity and signs of population expansion or selection in all the genes, including NP (Hd = 0.877 ± 0.00291), VP35 (0.904 ± 0.00090), VP40 (0.793 ± 0.00544), GP (0.938 ± 0.00116), VP30 (0.791 ± 0.00536), VP24 (0.631 ± 0.00791), and the L protein (0.978 ± 0.00030). Tajima’s D, Fu and Li’s D, and Fu and Li’s F values were all statistically significant (*p* < 0.05). Other MARV populations, including those from Angola, Sierra Leone, and the Uganda bat sub-population, did not exhibit statistically significant values for Tajima’s D, Fu and Li’s D, or Fu and Li’s F, with the exception of the Uganda human sub-population, which showed statistically significant negative Fu and Li’s D values for all genes, and Fu Li’s F values for VP40 and VP24 genes (*p* < 0.05). Overall, the Angola population displayed the lowest haplotype diversity, while the Uganda bat sub-population exhibited the highest haplotype diversity across all genes (Table 1).

For the RAVV population, no statistically significant neutrality indices were observed. In the Kenya population, which includes isolates from 1987, all the sequences shared the same haplotype across the NP, VP35, VP40, VP30, VP24, and L genes, showing no haplotype diversity, whereas the GP gene displayed two haplotypes with a moderate haplotype diversity (Hd = 0.667 ± 0.04167) (Table 2).

**Figure 1:**
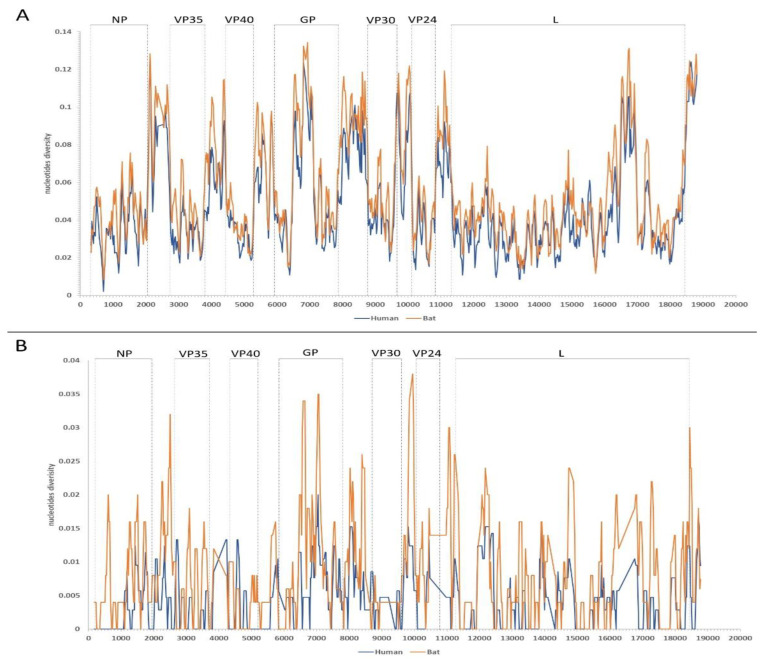
Sliding window analysis of (**A**) MARV and (**B**) RAVV genome sequences. Regions marked with dot lines are protein-coding regions labeled with their corresponding gene names. The following gene designations are used: NP (Nucleoprotein), VP35 (Viral Protein 35), VP40 (Viral Protein 40), GP (Glycoprotein), VP30 (Viral Protein 30), VP24 (Viral Protein 24), and L (RNA-dependent RNA polymerase).

The sliding window analysis of MARV and RAVV genomes revealed a variable nucleotide diversity across protein-coding regions. In the MARV genome, a lower nucleotide diversity was observed in the NP, VP35, VP40, VP30, and VP24 genes, with values ranging between 0.025 and 0.08, indicating these regions are highly conserved. In contrast, the GP and L genes exhibited a higher nucleotide diversity, with several regions exceeding 0.13. When analyzed by the host source, MARV isolates from human hosts showed a lower nucleotide diversity compared to those from bat hosts. Additionally, intergenic regions displayed greater diversity than protein-coding regions (Figure 1A).

In the RAVV genome, the nucleotide diversity by host source was inconsistent, with neither human nor bat isolates consistently displaying a higher diversity across the genome. However, certain protein-coding regions in bat isolates exhibited a considerably higher diversity compared to those from human isolates. For instance, human-related sequences showed a maximum nucleotide diversity of 0.02, whereas bat-related sequences exhibited a diversity exceeding 0.03 (Figure 1B). Comparatively, the MARV and RAVV genomes isolated from bats showed a higher nucleotide diversity across the protein-coding regions than the genomes isolated from human (Supplementary File S2: Figures S1–S14).

### 3.2. Phylogenetic Analysis

**Figure 2:**
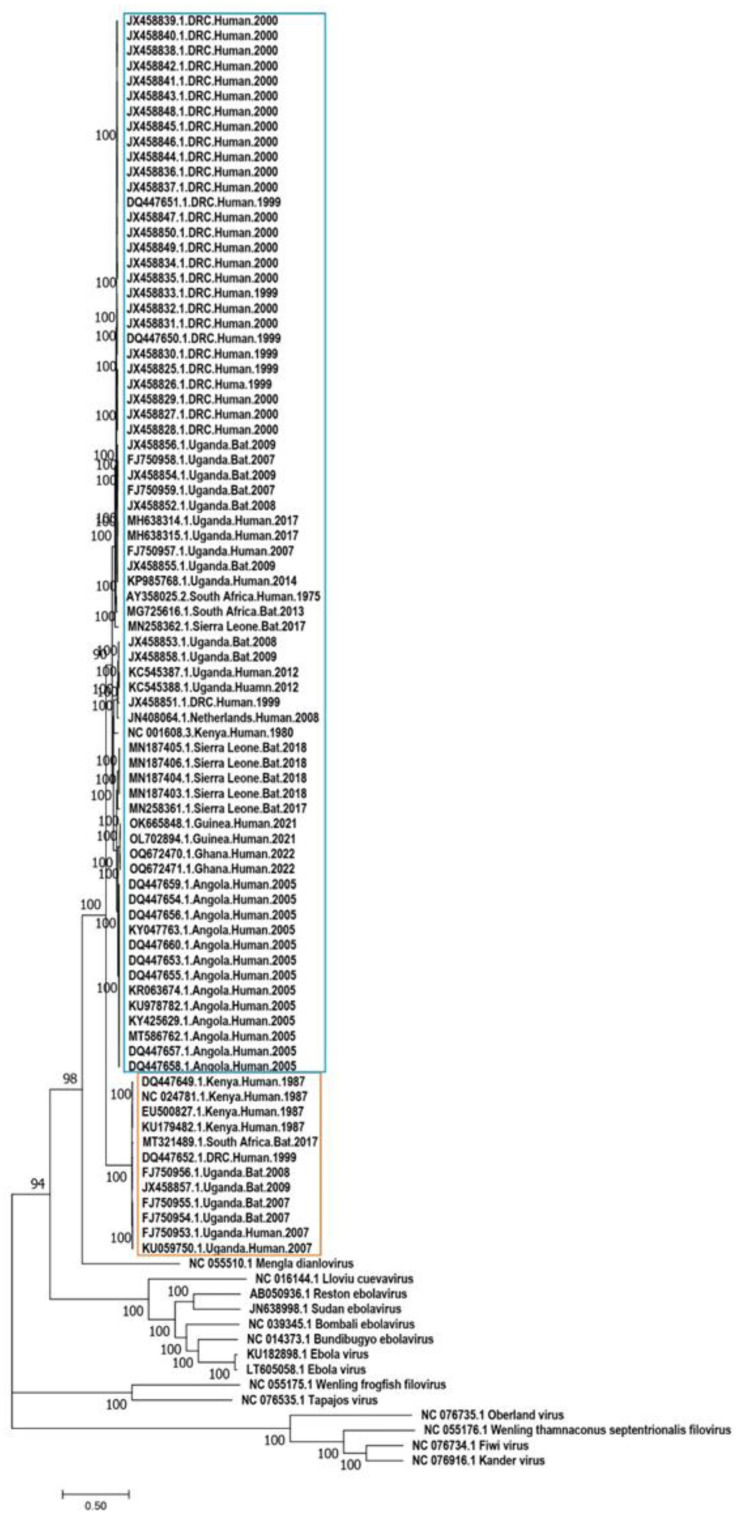
Maximum-likelihood phylogenetic tree. The phylogenetic tree of MARV and RAVV genomic sequences alongside reference genome sequences from other viruses within the Filoviridae family which was used as outgroup taxa. The blue-colored box highlights MARV sequences, while the beige-colored box highlights RAVV sequences. Bootstrap values supporting the clustering of branches are indicated next to the corresponding branches.

The phylogenetic analysis of MARV and RAVV genomic sequences revealed a clear clustering into two distinct clades when sequences from other genera within the Filoviridae family were incorporated (Figure 2).

**Figure 3:**
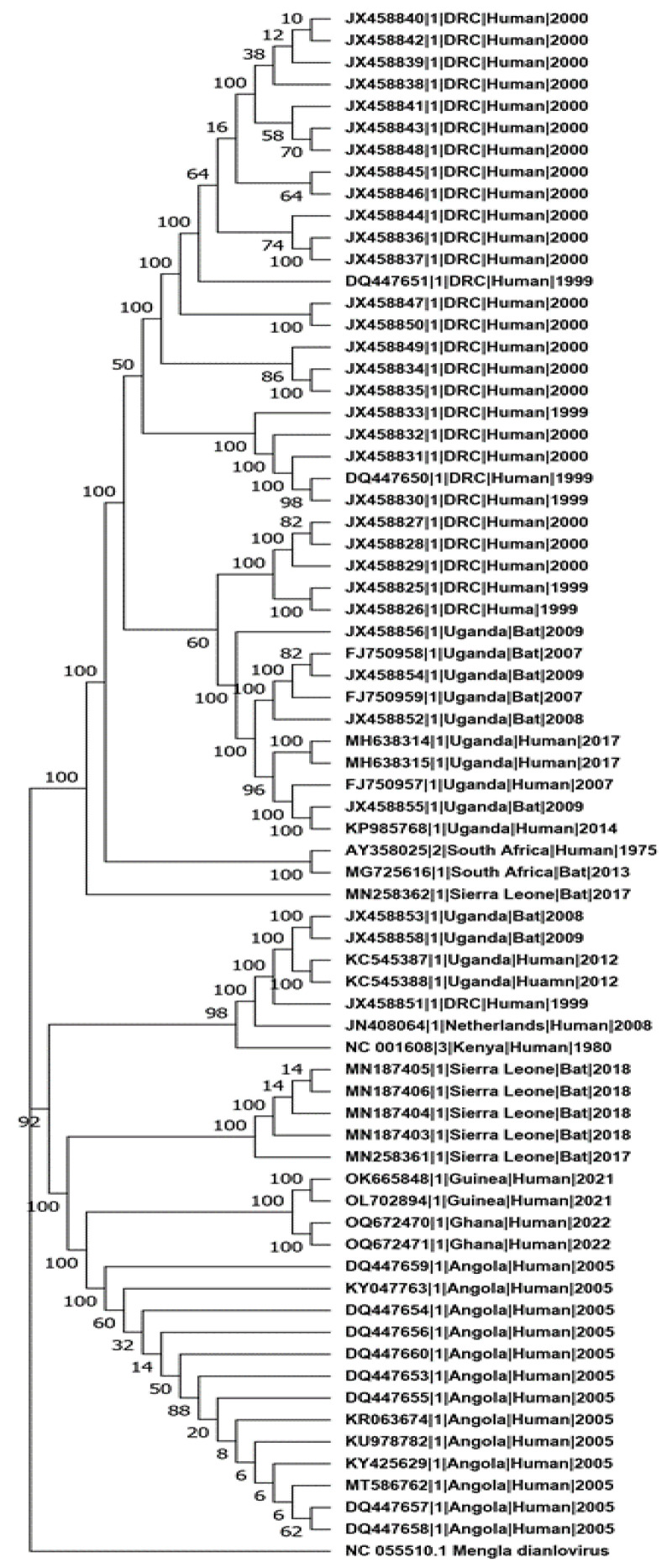
MARV maximum-likelihood phylogenetic tree. Bootstrap values supporting the clustering of branches are indicated next to the corresponding branches. *Mengla dianlovrius* reference sequence is used as outgroup taxon.

The MARV clade comprised two main lineages. One lineage primarily consisted of sequences from the 1998–2000 DRC outbreak and sequences reported from Uganda. Within this lineage, sequences from Uganda showed close relatedness, clustering with strong bootstrap support of 100%. Additionally, sequences from Sierra Leone and South Africa, originating from both human and bat hosts, indicated an older divergence within this lineage. The second MARV lineage included sequences also from humans and bats across Uganda, DRC, Kenya, Sierra Leone, Guinea, Ghana, and Angola, supported by a 92% bootstrap value. This lineage was further divided into three sub-lineages, each with strong bootstrap support of 98% or higher. One sub-lineage comprised sequences exclusively from bats in Sierra Leone collected during 2017–2018, another sub-lineage included sequences related to the 2021–2022 outbreak in Guinea and Ghana, and the third sub-lineage was exclusively a cluster of sequences from the 2005 Angola outbreak (Figure 3).

**Figure 4:**
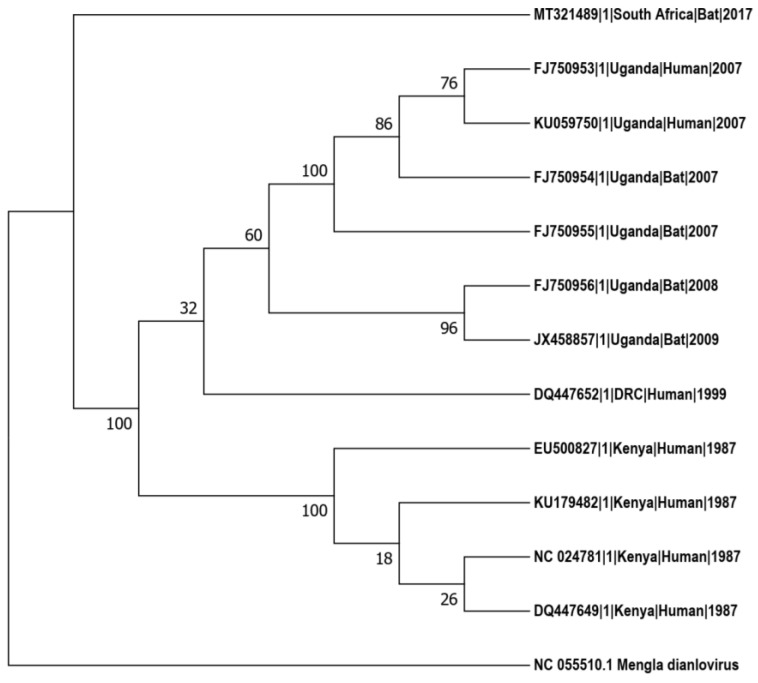
RAVV maximum-likelihood phylogenetic tree. Bootstrap values supporting the clustering of branches are indicated next to the corresponding branches. *Mengla dianlovrius* reference sequence is used as outgroup taxon.

In contrast, the RAVV clade displayed a single lineage that clustered sequences from both humans and bats, limited to Kenya, DRC, and Uganda. Notably, a South African sequence isolated from a bat in 2017 showed substantial divergence from the common ancestor of the RAVV clade and did not cluster within it, indicating significant genetic distance from related sequences. Additionally, RAVV sequences isolated in 1987 clustered separately from those isolated between 1999 and 2009, highlighting the distinct evolutionary groupings within this clade (Figure 4).

Moreover, when constructing phylogenetic trees for each gene separately in MARV and RAVV sequences to investigate their phylogenetic topologies, we observed consistent topologies across all genes. This uniformity in tree structure indicates that the genes of MARV and RAVV have diversified independently of each other, reinforcing the distinction between these two viruses at the genetic level (Supplementary File S2: Figures S15–S21).

### 3.3. Selection Pressure and Adaptive Evolution Analysis

The analysis of the SS/NSS ratio for the protein-coding regions of MARV and RAVV isolated from humans and bats showed similar ratios across the different host types. All SS/NSS ratios were below 1. The Dxy values, representing average nucleotide differences between the human and bat populations, showed the highest divergence in the GP gene in MARV and RAVV sequences, while the VP24 gene showed the least divergence in MARV sequences, and the VP40 gene in RAVV sequences. The Da values, which account for the within-population diversity, are consistently low across all genes. The number of shared mutations in MARV and RAVV sequences between the human and bat populations was the highest in the L gene, while the VP24 gene has the fewest shared mutations (Table 3).

Considering the minimum number of recombination events (Rms) observed for genes of MARV and RAVV across human and bat populations, MARV genes exhibited considerable recombination in both populations, with generally higher Rm values in humans compared to bats. For instance, the L gene showed the highest recombination, with 147 events in humans and 109 in bats, suggesting greater genetic exchange in the human host environment. Similarly, genes such as NP and GP demonstrate high recombination frequencies in humans (41 and 68 events, respectively) relative to bats (32 and 60 events). However, some MARV genes, such as VP30, show slightly more recombination in bats than in humans, indicating the variability in host influence on recombination rates. Conversely, RAVV genes exhibited no recombination events in either population (Table 3).

### 3.4. Haplotype Networks Analysis

**Figure 5:**
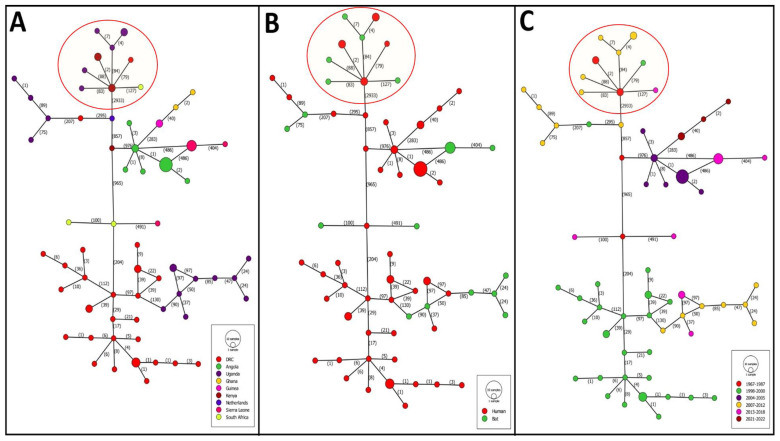
Minimum-spanning haplotype networks of MARV and RAVV genomic sequences. (**A**) This represents distribution of haplotypes according to countries of isolation. (**B**) This represents distribution of haplotypes according to host of isolation. (**C**) This represents distribution of haplotypes according to outbreaks years. The number of mutations between each haplotype is indicated between parentheses. Red circles represent haplotypes related to RAVV sequences.

The analysis of MARV haplotypes based on country, year, and host of isolation revealed a notable clustering of sequences from the DRC closely related to those from Uganda. This suggests a genetic connection between the two regions, likely maintained during outbreaks across different time periods, particularly the 1998–2000 outbreak in the DRC and the 2007–2012 and 2013–2018 outbreaks in Uganda. MARV haplotypes displayed complex genetic connectivity, with a range of 1 to 976 mutation steps, further supported by the inclusion of both human and bat isolates within these clusters. RAVV haplotypes, however, were distinctly separated from MARV haplotypes by 2933 mutation steps. Within the RAVV haplogroup, the highest number of mutation steps was observed between Kenya and South Africa, with 127 mutation steps. In contrast, within MARV haplotypes, Kenya and Angola exhibited 976 mutation steps, and Kenya and South Africa showed 965 mutation steps, underscoring the substantial genetic divergence among MARV haplotypes. This wide range of mutational steps further emphasizes the extensive genetic diversity within the MARV population (Figure 5).

The constructed haplotype networks for the MARV and RAVV genomes, with each gene analyzed separately, revealed varying levels of genetic divergence among genes. The L gene exhibited the highest number of mutational steps between MARV and RAVV haplotypes, while VP24 had the lowest. The number of mutational steps observed for each gene were as follows: NP (284), VP35 (135), VP40 (104), GP (382), VP30 (77), VP24 (42), and L (853). Shared haplotypes based on the region of virus isolation were observed only between Ghana and Guinea, specifically in the MARV VP24, VP30, and GP genes. Additionally, shared haplotypes between human and bat hosts were noted in MARV and RAVV VP24 and VP30 genes, and in RAVV GP, VP35, and VP40 genes (Supplementary File S2, Figures S22–S28).

## 4. Discussion

In this study, the observed co-circulation of MARV and RAVV within the same MVD outbreak periods, specifically during the 1998–2000 MVD outbreak in the DRC and the 2007 MVD outbreak in Uganda, underscores the importance of genomic surveillance, which aids in accurate virus identification, especially since both viruses cause similar clinical presentations [4,32].

Uniquely, this study analyzes the genetic diversity, phylodynamics, and population structure of MARV and RAVV independently, unlike previous studies that grouped them together. It also excludes recombinant strains and guinea-pig-adapted viruses, which artificially inflate the perceived genetic diversity due to adaptation for the mouse host [3,33,34]. Our findings highlight the separate evolutionary trajectories for MARV and RAVV where MARV lineages exhibit a within-lineage diversity while maintaining distinct clustering, suggesting limited recombination or genetic exchange. In contrast, RAVV sequences show an absence of recombination, indicative of a stable evolutionary path with preserved inheritance patterns across genomic regions, reinforcing a structured evolutionary framework. Although it is important to interpret the results for RAVV with caution, as only twelve genome sequences were available for this analysis, even when these sequences were treated as a single population regardless of host type, no evidence of recombination was found across the RAVV genes. This finding underscores the evolutionary distinctness and stability of RAVV relative to MARV, supporting the notion that MARV and RAVV are distinct entities with unique evolutionary patterns [35].

The phylogenetic analysis revealed that MARV is organized into two primary lineages, each containing several sub-lineages associated with specific geographic regions and host species. This clustering differs slightly from previous studies, which identified three lineages [34]; however, the two-lineage organization in our analysis was strongly supported. This support was consistent not only at the whole-genome level but also across individual gene analyses, with nearly identical tree topologies. Consequently, we concluded that the two main clusters represent the primary lineages, while clustering within each lineage represents sub-lineages. This organization aligns with evidence suggesting MARV diversification in response to regional and ecological factors [36]. The structured presence of these sub-lineages also points to a shared ancestral MARV population maintained through cross-host transmission, highlighting the virus’s adaptability and its capacity to persist across various ecological environments [34]. These sub-lineages exhibit limited genetic divergence and specific geographic regions and host species clustering, pointing to a shared ancestral population that has persisted over time rather than arising from repeated independent spillover events. Notably, the unique sub-lineage clustering of bat-derived sequences from the Sierra Leone epidemiological field study (2017–2018) [13] and their separation from those isolated from bats in Uganda (2008–2009) [37,38] indicate that regional ecological or geographic factors may drive MARV evolution in bat populations. This distinct clustering suggests recent ecological diversification, with MARV potentially adapting to specific local environments, which could influence its transmission dynamics and persistence in certain regions. A similar pattern was observed for MARV sequences from the 2005 Angola outbreak, which included only human-derived isolates [3]. Their phylogenetic distinctness points to a recent divergence event likely contributing to evolutionary changes that may have enhanced viral transmissibility and virulence.

Additionally, a haplotype network analysis revealed regional genetic connectivity suggesting transmission dynamics, particularly between the DRC and Uganda, where DRC sequences from the 1998–2000 outbreak closely cluster with Ugandan sequences from 2007–2012 and 2013–2018 [5,39,40,41]. Such clustering suggests a persistent transmission link between these regions, possibly facilitated by cross-border viral transmission sustained by shared ecological conditions or cross-species transmission. This pattern may also indicate strains adapted for transmissibility, potentially serving as progenitors of various lineages. Further investigation into whether MARV and RAVV are evolving toward specific haplotypes associated with higher fitness or epidemic potential is essential.

The reduced genetic diversity observed in Angola isolates suggests that MARV underwent adaptation to survive in varied environmental conditions and host reservoirs. Such adaptation is likely influenced by selective pressures in the new ecology, which could include host immune responses, or other factors unique to each region [42]. These adaptations may contribute to the virus’s fitness and persistence across diverse habitats and hosts. Notably, the MVD outbreak in Angola caused by MARV recorded a case fatality rate of approximately 90%—higher than other documented MVD outbreaks—and saw extensive spread, resulting in 252 reported cases and 227 deaths [3].

The observed relationship between Angola isolates and the Kenya Musoke strain, as well as isolates from West African countries like Sierra Leone, Guinea, and Ghana, provides compelling evidence for the cross-regional transmission of MARV. This genetic connectivity across haplotypes suggests potential transboundary events in which the virus may have spread between East and West Africa, likely facilitated by known bat migration patterns and wildlife corridors [43]. This movement also hints at a possible attenuation of virulence as MARV adapts to different ecological niches. Such reduced virulence may result from a trade-off in the virus’s replication dynamics, where high transmissibility or adaptation to new hosts and environments coincides with milder pathogenic effects. This hypothesis aligns with the relatively low death tolls reported in Guinea and Ghana, with only one and two deaths, respectively. For instance, in Ghana, 110 cases were reported, which may indicate viral adaptation to local host populations, while, in Guinea, early detection and a rapid response limited the outbreak to a single confirmed case [9,44,45]. These patterns suggest the virus may have become less lethal but more sustainable in certain regions or host populations. Additionally, these findings underscore how localized factors can drive MARV’s genetic divergence, shaping its epidemiology and virulence during outbreaks.

The neutrality testing of MARV populations revealed distinct genetic variability by region and host, suggesting varied evolutionary pressures. The high haplotype diversity in DRC populations points to selective pressures that may enhance viral adaptability, supported by significant neutrality values in protein-coding regions. In Uganda, MARV sequences from humans showed purifying selection, indicating adaptation to the human immune system, while bat-derived sequences displayed a high diversity without significant neutrality values, suggesting bats as reservoirs that contribute to MARV’s genetic diversity with minimal immune-driven structural changes [38,46,47]. A sliding window analysis highlighted conserved protein-coding regions, particularly NP, VP35, VP40, VP30, and VP24, which undergo purifying selection for essential viral functions, enabling immune evasion by introducing minor changes that prevent immune recognition while preserving replication efficiency [48,49,50,51]. Low synonymous-to-non-synonymous substitution ratios for both MARV and RAVV isolates indicate strong purifying selection, preserving protein function. The divergence in the GP gene suggests the adaptation to host-specific interactions, while VP24’s stability underscores its role in immune evasion across hosts [33]. The genes with shared haplotypes likely contribute to viral fitness across species, enabling MARV or RAVV to jump between bat reservoirs and humans. VP24 and VP30 are key in immune evasion and viral replication, potentially making them vital for adapting to diverse host environments [52,53,54].

In the current ongoing MVD outbreak in Rwanda, the open borders with DRC and Tanzania, where MVD outbreaks had occurred recently, suggest the possibility of cross-border spillover or human-to-human transmission from the DRC. MARV’s diversity appears to be driven by geographic and temporal factors, underscoring its evolutionary adaptability across Africa. This continuity also supports the role of viral reservoirs in MARV persistence in Central Africa, especially within the DRC [2]. Therefore, countries at risk of the diseases should invest in strengthening Global Health Security through institutionalizing the implementation of a transdisciplinary multisectoral One Health strategy [12,15]. Implementing a national One Health prioritization exercise will help in identifying zoonotic diseases of public health importance [55,56,57,58,59]. Then, in collaboration with local and international stakeholders of One Health, these diseases of high priority could be jointly targeted with an integrated One Health preparedness, prevention, surveillance, and response strategy to enhance the cost-effectiveness and reduce the associated labor and required time for planning and implementation [12,56,58,59,60,61]. This will enhance Global Health Security through the cross-border coordination and joint implementation of the International Health Regulations (IHRs 2005) [62,63,64].

## 5. Conclusions

It seems that, driven by the high dynamics and rapid and distant movements of the main reservoir, fruit bats, Marburg and Ravn viruses are rapidly evolving and adapting to transmission among the human population. This growing adaptability might be attributed to several factors including increased undetected transmission, expansion in the host range, and changes in climate and land use and cover as well as human activities, which increase the contact between a wide range of animal species including humans. More investigations in holistic One Health are needed to explore these hypotheses and provide up-to-date evidence to inform policymaking, strategic planning, and the development and implementation of cost-effective interventions for the prevention and control of the diseases.

## Figures and Tables

**Table 1:** Neutrality testing and genetic diversity analysis of MARV population.

Population ¥	n	S	Eta	Hap	Hd ± VarHd	Pi	AvDif	Tajima D	Fu Li’s D	Fu Li’s F
NP gene										
DRC Human	29	139	139	15	0.877 ± 0.00291	0.00690	14.399	−2.2949 *	−1.0584 *	−1.0549 *
Uganda	14	142	142	12	0.978 ± 0.00119	0.02646	55.242	1.0635	−0.5462	−0.4411
Uganda Human	6	123	123	4	0.867 ± 0.01667	0.03078	64.267	1.2496	−0.8115 *	−0.8151
Uganda Bat	8	131	131	8	1.000 ± 0.00391	0.02578	53.821	0.3562	−0.6933	−0.6646
Angola Human	13	3	3	4	0.423 ± 0.02705	0.00022	0.4620	−1.6523	−0.5802	−0.4856
Sierra Leone Bat	6	137	138	3	0.600 ± 0.04630	0.02382	49.733	−1.1475	−0.8115	−0.8151
VP35 gene										
DRC Human	29	74	74	13	0.904 ± 0.00090	0.00834	8.2560	−2.1397 *	−0.9686 *	−0.9789 *
Uganda	14	74	74	11	0.967 ± 0.00134	0.02831	28.022	0.9057	−0.4641	−0.339
Uganda Human	6	61	61	4	0.867 ± 0.01667	0.03226	31.933	1.2554	−0.8222 *	−0.8196
Uganda Bat	8	69	69	7	0.964 ± 0.00596	0.02796	27.679	0.2171	−0.7133	−0.6769
Angola Human	13	1	1	2	0.154 ± 0.01590	0.00016	0.1540	−1.1491	−0.5113	−0.3981
Sierra Leone Bat	6	74	74	3	0.600 ± 0.04630	0.02714	26.867	−1.1019	−0.8222	−0.8196
VP40 gene										
DRC Human	29	63	63	12	0.793 ± 0.00544	0.00775	7.0640	−2.1191 *	−1.4735 *	−1.4003 *
Uganda	14	65	65	10	0.945 ± 0.00203	0.02762	25.187	1.0267	−0.981	−0.9095
Uganda Human	6	56	56	4	0.867 ± 0.01667	0.03231	29.467	1.2932	−0.8309 *	−0.8295 *
Uganda Bat	8	63	63	6	0.893 ± 0.01238	0.02710	24.714	0.0927	−0.8673	−0.8455
Angola Human	13	2	2	2	0.154 ± 0.01590	0.00034	0.3080	−1.468	−0.9568	−0.8893
Sierra Leone Bat	6	66	66	3	0.600 ± 0.04630	0.02588	23.600	−1.1809	−0.8309	−0.8295
GP gene										
DRC Human	29	227	228	19	0.938 ± 0.00116	0.01339	27.387	−2.0584 *	−1.2524 *	−1.2432 *
Uganda	14	227	228	12	0.978 ± 0.00119	0.04304	88.066	1.0288	−0.6956	−0.6145
Uganda Human	6	200	200	4	0.867 ± 0.01667	0.05103	104.40	1.2458	−0.7361 *	−0.7385
Uganda Bat	8	202	202	8	1.000 ± 0.00391	0.04109	84.071	0.4332	−0.7492	−0.7333
Angola Human	13	2	2	3	0.410 ± 0.02368	0.00021	0.4360	−0.9092	−0.7286	−0.6594
Sierra Leone Bat	6	235	240	3	0.600 ± 0.04630	0.04148	84.867	−1.2512	−0.7361	−0.7385
VP30 gene										
DRC Human	29	78	79	12	0.791 ± 0.00536	0.01013	8.574	−2.1897 *	−1.2083 *	−1.1940 *
Uganda	14	72	72	10	0.956 ± 0.00142	0.03245	27.451	0.9415	−0.6712	−0.582
Uganda Human	6	63	63	4	0.867 ± 0.01667	0.03901	33.000	1.2605	−0.7312 *	−0.7321
Uganda Bat	8	63	63	7	0.964 ± 0.00596	0.03052	25.821	0.339	−0.7751	−0.7606
Angola Human	13	2	2	3	0.295 ± 0.02427	0.00036	0.3080	−1.468	−0.6713	−0.5888
Sierra Leone Bat	6	72	72	3	0.600 ± 0.04630	0.03002	25.400	−1.2528	−0.7312	−0.7321
VP24 gene										
DRC Human	29	40	40	6	0.631 ± 0.00791	0.00469	3.571	−2.4061 *	−0.5604 *	−0.5087 *
Uganda	14	53	53	11	0.956 ± 0.00200	0.02620	19.967	0.8704	−0.428	−0.3414
Uganda Human	6	42	42	4	0.867 ± 0.01667	0.02913	22.200	1.3213	−0.6335 *	−0.6309 *
Uganda Bat	8	50	50	8	1.000 ± 0.00391	0.02643	20.143	0.2397	−0.5522	−0.5231
Angola Human	13	1	1	2	0.513 ± 0.00675	0.00067	0.5130	1.3005	−0.5227	−0.4566
Sierra Leone Bat	6	43	43	3	0.600 ± 0.04630	0.01986	15.133	−1.2549	−0.6335	−0.6309
L gene										
DRC Human	29	524	525	23	0.978 ± 0.00030	0.00807	56.461	−2.2653 *	−1.2662 *	−1.2593 *
Uganda	14	569	576	12	0.978 ± 0.00119	0.03116	218.022	0.9222	−0.6498	−0.5472
Uganda Human	6	488	490	4	0.867 ± 0.01667	0.03652	255.467	1.2396	−0.8458 *	−0.8499
Uganda Bat	8	518	522	8	1.000 ± 0.00391	0.03005	210.25	0.2436	−0.763	−0.7373
Angola Human	13	4	4	4	0.603 ± 0.01707	0.00014	0.9740	−0.8291	−0.6811	−0.5894
Sierra Leone Bat	6	569	570	3	0.600 ± 0.04630	0.02901	202.933	−1.218	−0.8458	−0.8499

DRC: Democratic Republic of Congo, n: number of sequences, S: segregating sites, Eta: number of mutations, Hap: number of haplotypes, Hd ± VarHd: haplotype diversity ± variance of haplotype diversity, Pi: nucleotide diversity, AvDif: average number of nucleotide differences. ¥ Populations with fewer than 3 sequences, including those from Ghana (2), Guinea (2), The Netherlands (1), Kenya (1), and South Africa (2), were excluded from the analysis to reduce bias caused by small sample size. * indicates statistical significance, with *p*-values < 0.05.

**Table 2:** Neutrality testing and genetic diversity analysis of RAVV population.

Population ¥	n	S	Eta	Hap	Hd ± VarHd	Pi	AvDif	Tajima D	Fu Li’s D	Fu Li’s F
NP gene										
Uganda	6	17	17	4	0.867 ± 0.01667	0.00300	6.26670	−0.9819	−1.3715	−1.3729
Uganda Bat	4	16	16	3	0.833 ± 0.04948	0.00399	8.33330	−0.4599	−0.6826	−0.6826
Kenya Human	4	0	0	1	0.000 ± 0.00000	0.00000	0.00000	n.d.	n.d.	n.d.
VP35 gene										
Uganda	6	10	10	4	0.800 ± 0.02963	0.00397	3.93330	−0.6119	−1.51	−1.5076
Uganda Bat	4	10	10	4	1.000 ± 0.03125	0.00556	5.50000	0.0834	−0.8473	−0.8473
Kenya Human	4	0	0	1	0.000 ± 0.00000	0.00000	0.00000	n.d.	n.d.	n.d.
VP40 gene										
Uganda	6	1	1	2	0.533 ± 0.02963	0.00058	0.53330	0.8506	−1.3999	−1.3979
Uganda Bat	4	1	1	2	0.667 ± 0.04167	0.00073	0.66670	1.633	−0.8086	−0.8086
Kenya Human	4	0	0	1	0.000 ± 0.00000	0.00000	0.00000	n.d.	n.d.	n.d.
GP gene										
Uganda	6	31	31	3	0.600 ± 0.04630	0.00661	13.5333	−0.0202	−1.3024	−1.3053
Uganda Bat	4	31	31	3	0.833 ± 0.04948	0.00888	18.1667	0.7693	−0.6143	−0.6143
Kenya Human	4	1	1	2	0.667 ± 0.04167	0.00033	0.6667	1.633	−0.6143	−0.6143
VP30 gene										
Uganda	6	5	5	3	0.600 ± 0.04630	0.00244	2.06670	−0.3147	−1.3245	−1.3228
Uganda Bat	4	5	5	3	0.833 ± 0.04948	0.00335	2.83330	0.3719	−0.7801	−0.7801
Kenya Human	4	0	0	1	0.000 ± 0.00000	0.00000	0.00000	n.d.	n.d.	n.d.
VP24 gene										
Uganda	6	2	2	3	0.600 ± 0.04630	0.00087	0.66670	−1.132	−1.4239	−1.4219
Uganda Bat	4	2	2	3	0.833 ± 0.04948	0.00131	1.00000	−0.7099	−0.8173	−0.8173
Kenya Human	4	0	0	1	0.000 ± 0.00000	0.00000	0.00000	n.d.	n.d.	n.d.
L gene										
Uganda	6	72	72	5	0.933 ± 0.01481	0.00418	29.2000	−0.4765	−1.5064	−1.5028
Uganda Bat	4	71	71	4	1.000 ± 0.03125	0.00568	39.6667	0.2532	−0.834	−0.834
Kenya Human	4	0	0	1	0.000 ± 0.00000	0.00000	0.00000	n.d.	n.d.	n.d.

DRC: Democratic Republic of Congo, n: number of sequences, S: segregating sites, Eta: number of mutations, Hap: number of haplotypes, Hd ± VarHd: haplotype diversity ± variance of haplotype diversity, Pi: nucleotide diversity, AvDif: average number of nucleotide differences. ¥ Populations with fewer than 3 sequences, including those from South Africa (1), DRC (1), and Uganda Human isolate (2) were excluded from the analysis to reduce bias caused by small sample size. n.d. means not determine.

**Table 3:** Analysis of synonymous to non-synonymous substitution ratios for the protein-coding regions of MARV isolated based on host of isolation.

Protein-Coding Region	SS/NSS Ratio		Dxy	Da	Shared Mutations	Rm	
	Human	Bat				Human	Bat
MARV							
NP	0.295	0.295	0.03839	0.00141	182	41	32
VP35	0.308	0.311	0.03826	0.00143	83	24	11
VP40	0.303	0.302	0.03772	0.00160	70	24	17
GP	0.306	0.306	0.05850	0.00171	226	68	60
VP30	0.292	0.293	0.04397	0.00121	81	11	13
VP24	0.286	0.284	0.03685	0.00182	58	13	7
L	0.290	0.291	0.04304	0.00220	648	147	109
RAVV							
NP	0.292	0.292	0.00486	0.00047	3	0	0
VP35	0.300	0.300	0.00494	0.00004	3	0	0
VP40	0.312	0.312	0.00305	0.00055	1	0	0
GP	0.300	0.305	0.00882	0.00009	14	0	0
VP30	0.291	0.298	0.00409	0.00100	1	0	0
VP24	0.283	0.278	0.00505	0.00075	0	0	0
L	0.293	0.295	0.00562	0.00027	21	0	0

SS: synonymous substitution, NSS: non-synonymous substitutions, Dxy: average number of nucleotide differences per site between populations, Da: net nucleotide differences, Rm: minimum number of recombination events.

## Data Availability

All data produced during this study are included in the published article and its Supplementary Files.

## Supplementary Materials

The following supporting information can be downloaded at: https://www.mdpi.com/article/10.3390/pathogens13121107/s1. File S1: Tables S1–S3; File S2: Figures S1–S28.
