# Supplementary material for: Genomic Evolution and Phylodynamics of the Species Orthomarburgvirus marburgense (Marburg and Ravn Viruses) to Understand Viral Adaptation and Marburg Virus Disease’s Transmission Dynamics
Source: Pathogens. 2024 Dec 14;13(12):1107. doi: 10.3390/pathogens13121107 (PMC11728648; doi:10.3390/pathogens13121107)
Supplement: Supplementary file 1 [file pathogens-13-01107-s001.zip › pathogens-3347639-supplementary/Supplementary File S1.pdf]

Supplementary File 1:

Table S1: Reference sequences of MARV and RAVV genomes excluded from the study

Accession No.	Marburg virus genus	Sequence length	Reason to exclude	Reference link
AF005730.1	MARV	1557	Single gene	https://www.ncbi.nlm.nih.gov/nuccore/MF939097.1
AF005731.1	MARV	1557	Single gene	https://www.ncbi.nlm.nih.gov/nuccore/KM261523.1
AF005732.1	MARV	1557	Single gene	https://www.ncbi.nlm.nih.gov/nuccore/KY047764.1
AF005733.1	MARV	2046	Single gene	https://www.ncbi.nlm.nih.gov/nuccore/OQ847644.1
AF005734.1	MARV	2046	Single gene	https://www.ncbi.nlm.nih.gov/nuccore/AY430365.1
AF005735.1	MARV	2046	Single gene	https://www.ncbi.nlm.nih.gov/nuccore/AY430366.1
AY430365.1	MARV	19113	Lab adapt isolate	https://www.ncbi.nlm.nih.gov/nuccore/Z12132.1
AY430366.1	MARV	19112	Lab adapt isolate	https://www.ncbi.nlm.nih.gov/nuccore/GQ433353.1
DQ466108.1	MARV	302	Partial gene	https://www.ncbi.nlm.nih.gov/nuccore/EF446132.1
DQ466110.1	MARV	302	Partial gene	https://www.ncbi.nlm.nih.gov/nuccore/Z29337.1
DQ466112.1	MARV	302	Partial gene	https://www.ncbi.nlm.nih.gov/nuccore/GQ433352.1
DQ466116.1	MARV	302	Partial gene	https://www.ncbi.nlm.nih.gov/nuccore/GQ433351.1
DQ466131.1	MARV	302	Partial gene	https://www.ncbi.nlm.nih.gov/nuccore/KP117261.1
DQ466135.1	MARV	302	Partial gene	https://www.ncbi.nlm.nih.gov/nuccore/KP117260.1
DQ466138.1	MARV	302	Partial gene	https://www.ncbi.nlm.nih.gov/nuccore/KP117259.1
DQ466143.1	MARV	302	Partial gene	https://www.ncbi.nlm.nih.gov/nuccore/MK271062.1
DQ466144.1	MARV	367	Partial gene	https://www.ncbi.nlm.nih.gov/nuccore/M92834.1
DQ466145.1	MARV	367	Partial gene	https://www.ncbi.nlm.nih.gov/nuccore/X68494.1
DQ466146.1	MARV	367	Partial gene	https://www.ncbi.nlm.nih.gov/nuccore/M72714.1
DQ466148.1	MARV	367	Partial gene	https://www.ncbi.nlm.nih.gov/nuccore/X68495.1
DQ466150.1	MARV	367	Partial gene	https://www.ncbi.nlm.nih.gov/nuccore/X64406.1
DQ466160.1	MARV	367	Partial gene	https://www.ncbi.nlm.nih.gov/nuccore/X68493.1
DQ466161.1	MARV	367	Partial gene	https://www.ncbi.nlm.nih.gov/nuccore/X64405.1
DQ466173.1	MARV	367	Partial gene	https://www.ncbi.nlm.nih.gov/nuccore/AF005735.1
DQ466174.1	MARV	245	Partial gene	https://www.ncbi.nlm.nih.gov/nuccore/DQ656106.1
DQ466175.1	MARV	245	Partial gene	https://www.ncbi.nlm.nih.gov/nuccore/DQ656107.1
DQ466176.1	MARV	245	Partial gene	https://www.ncbi.nlm.nih.gov/nuccore/AF005733.1
DQ466179.1	MARV	245	Partial gene	https://www.ncbi.nlm.nih.gov/nuccore/AF005730.1
DQ466181.1	MARV	245	Partial gene	https://www.ncbi.nlm.nih.gov/nuccore/AF005732.1
DQ466184.1	MARV	245	Partial gene	https://www.ncbi.nlm.nih.gov/nuccore/AF005734.1
DQ466186.1	MARV	450	Partial gene	https://www.ncbi.nlm.nih.gov/nuccore/AF005731.1
DQ466187.1	MARV	450	Partial gene	https://www.ncbi.nlm.nih.gov/nuccore/MT929358.1

DQ466188.1	MARV	450	Partial gene	https://www.ncbi.nlm.nih.gov/nuccore/MT929356.1
DQ466189.1	MARV	450	Partial gene	https://www.ncbi.nlm.nih.gov/nuccore/MT929357.1
DQ466191.1	MARV	450	Partial gene	https://www.ncbi.nlm.nih.gov/nuccore/LC465155.1
DQ466192.1	MARV	450	Partial gene	https://www.ncbi.nlm.nih.gov/nuccore/MF871598.1
DQ466193.1	MARV	449	Partial gene	https://www.ncbi.nlm.nih.gov/nuccore/MN193425.1
DQ466194.1	MARV	247	Partial gene	https://www.ncbi.nlm.nih.gov/nuccore/EF490232.1
DQ466195.1	MARV	250	Partial gene	https://www.ncbi.nlm.nih.gov/nuccore/OP716850.1
DQ656106.1	MARV	2046	Recombinant	https://www.ncbi.nlm.nih.gov/nuccore/LC465158.1
DQ656107.1	MARV	2046	Recombinant	https://www.ncbi.nlm.nih.gov/nuccore/MN193426.1
EF446132.1	RAVV	19112	Lab adapt isolate	https://www.ncbi.nlm.nih.gov/nuccore/EU068110.1
EF490232.1	RAVV	710	Partial gene	https://www.ncbi.nlm.nih.gov/nuccore/MN193424.1
EU068108.1	MARV	464	Partial gene	https://www.ncbi.nlm.nih.gov/nuccore/EU068108.1
EU068109.1	MARV	464	Partial gene	https://www.ncbi.nlm.nih.gov/nuccore/MN193427.1
EU068110.1	MARV	464	Partial gene	https://www.ncbi.nlm.nih.gov/nuccore/EU068109.1
EU068111.1	MARV	302	Partial gene	https://www.ncbi.nlm.nih.gov/nuccore/MN193419.1
EU068112.1	MARV	302	Partial gene	https://www.ncbi.nlm.nih.gov/nuccore/FJ743651.1
EU068113.1	MARV	302	Partial gene	https://www.ncbi.nlm.nih.gov/nuccore/FJ743655.1
EU118794.1	MARV	302	Partial gene	https://www.ncbi.nlm.nih.gov/nuccore/FJ743675.1
EU118795.1	MARV	302	Partial gene	https://www.ncbi.nlm.nih.gov/nuccore/FJ743642.1
EU118796.1	MARV	302	Partial gene	https://www.ncbi.nlm.nih.gov/nuccore/FJ743667.1
EU118798.1	MARV	302	Partial gene	https://www.ncbi.nlm.nih.gov/nuccore/JX462498.1
EU118799.1	MARV	302	Partial gene	https://www.ncbi.nlm.nih.gov/nuccore/FJ743653.1
EU118804.1	MARV	302	Partial gene	https://www.ncbi.nlm.nih.gov/nuccore/FJ743649.1
FJ743642.1	MARV	443	Partial gene	https://www.ncbi.nlm.nih.gov/nuccore/FJ743671.1
FJ743647.1	MARV	443	Partial gene	https://www.ncbi.nlm.nih.gov/nuccore/DQ466187.1
FJ743648.1	MARV	443	Partial gene	https://www.ncbi.nlm.nih.gov/nuccore/JX462500.1
FJ743649.1	MARV	443	Partial gene	https://www.ncbi.nlm.nih.gov/nuccore/DQ466193.1
FJ743651.1	MARV	443	Partial gene	https://www.ncbi.nlm.nih.gov/nuccore/GQ499199.1
FJ743653.1	MARV	443	Partial gene	https://www.ncbi.nlm.nih.gov/nuccore/JX462492.1
FJ743655.1	MARV	443	Partial gene	https://www.ncbi.nlm.nih.gov/nuccore/FJ743647.1
FJ743667.1	MARV	443	Partial gene	https://www.ncbi.nlm.nih.gov/nuccore/JX462497.1
FJ743668.1	MARV	443	Partial gene	https://www.ncbi.nlm.nih.gov/nuccore/JX462501.1
FJ743669.1	MARV	443	Partial gene	https://www.ncbi.nlm.nih.gov/nuccore/FJ743648.1
FJ743670.1	MARV	443	Partial gene	https://www.ncbi.nlm.nih.gov/nuccore/DQ466189.1

FJ743671.1	MARV	443	Partial gene	https://www.ncbi.nlm.nih.gov/nuccore/DQ466188.1
FJ743675.1	MARV	443	Partial gene	https://www.ncbi.nlm.nih.gov/nuccore/OL956937.1
FJ743676.1	MARV	443	Partial gene	https://www.ncbi.nlm.nih.gov/nuccore/OL956939.1
FJ743679.1	MARV	296	Partial gene	https://www.ncbi.nlm.nih.gov/nuccore/JX462494.1
FJ743681.1	MARV	296	Partial gene	https://www.ncbi.nlm.nih.gov/nuccore/DQ466192.1
FJ743683.1	MARV	296	Partial gene	https://www.ncbi.nlm.nih.gov/nuccore/DQ466186.1
FJ743685.1	MARV	296	Partial gene	https://www.ncbi.nlm.nih.gov/nuccore/MN193420.1
GQ433351.1	MARV	19114	Lab adapt isolate	https://www.ncbi.nlm.nih.gov/nuccore/DQ466191.1
GQ433352.1	MARV	19113	Lab adapt isolate	https://www.ncbi.nlm.nih.gov/nuccore/JX462502.1
GQ433353.1	MARV	19113	Lab adapt isolate	https://www.ncbi.nlm.nih.gov/nuccore/FJ743668.1
GQ499199.1	MARV	464	Partial gene	https://www.ncbi.nlm.nih.gov/nuccore/MN193422.1
JQ272621.1	MARV	302	Partial gene	https://www.ncbi.nlm.nih.gov/nuccore/JX462493.1
JX462491.1	MARV	404	Partial gene	https://www.ncbi.nlm.nih.gov/nuccore/MN193423.1
JX462492.1	MARV	443	Partial gene	https://www.ncbi.nlm.nih.gov/nuccore/MN193421.1
JX462493.1	MARV	412	Partial gene	https://www.ncbi.nlm.nih.gov/nuccore/KJ747229.1
JX462494.1	MARV	435	Partial gene	https://www.ncbi.nlm.nih.gov/nuccore/DQ466160.1
JX462497.1	MARV	440	Partial gene	https://www.ncbi.nlm.nih.gov/nuccore/DQ466150.1
JX462498.1	MARV	440	Partial gene	https://www.ncbi.nlm.nih.gov/nuccore/DQ466161.1
JX462499.1	MARV	440	Partial gene	https://www.ncbi.nlm.nih.gov/nuccore/KJ747213.1
JX462500.1	MARV	440	Partial gene	https://www.ncbi.nlm.nih.gov/nuccore/DQ466146.1
JX462501.1	MARV	440	Partial gene	https://www.ncbi.nlm.nih.gov/nuccore/DQ466173.1
JX462502.1	MARV	447	Partial gene	https://www.ncbi.nlm.nih.gov/nuccore/KJ747212.1
JX462503.1	MARV	284	Partial gene	https://www.ncbi.nlm.nih.gov/nuccore/DQ466144.1
JX462505.1	MARV	313	Partial gene	https://www.ncbi.nlm.nih.gov/nuccore/KJ747220.1
JX462506.1	MARV	325	Partial gene	https://www.ncbi.nlm.nih.gov/nuccore/DQ466148.1
JX462507.1	MARV	325	Partial gene	https://www.ncbi.nlm.nih.gov/nuccore/KJ747215.1
KJ747211.1	MARV	332	Partial gene	https://www.ncbi.nlm.nih.gov/nuccore/DQ466145.1
KJ747212.1	MARV	367	Partial gene	https://www.ncbi.nlm.nih.gov/nuccore/KJ747214.1
KJ747213.1	MARV	367	Partial gene	https://www.ncbi.nlm.nih.gov/nuccore/KJ747216.1
KJ747214.1	MARV	361	Partial gene	https://www.ncbi.nlm.nih.gov/nuccore/KJ747217.1
KJ747215.1	MARV	367	Partial gene	https://www.ncbi.nlm.nih.gov/nuccore/KJ747218.1
KJ747216.1	MARV	366	Partial gene	https://www.ncbi.nlm.nih.gov/nuccore/KJ747227.1
KJ747217.1	MARV	365	Partial gene	https://www.ncbi.nlm.nih.gov/nuccore/KJ747224.1
KJ747218.1	MARV	365	Partial gene	https://www.ncbi.nlm.nih.gov/nuccore/KJ747226.1

KJ747219.1	MARV	365	Partial gene	https://www.ncbi.nlm.nih.gov/nuccore/KJ747222.1
KJ747220.1	MARV	365	Partial gene	https://www.ncbi.nlm.nih.gov/nuccore/KJ747223.1
KJ747222.1	MARV	370	Partial gene	https://www.ncbi.nlm.nih.gov/nuccore/KJ747219.1
KJ747223.1	MARV	351	Partial gene	https://www.ncbi.nlm.nih.gov/nuccore/KJ747234.1
KJ747224.1	MARV	356	Partial gene	https://www.ncbi.nlm.nih.gov/nuccore/KJ747232.1
KJ747225.1	MARV	323	Partial gene	https://www.ncbi.nlm.nih.gov/nuccore/KJ747228.1
KJ747226.1	MARV	355	Partial gene	https://www.ncbi.nlm.nih.gov/nuccore/MN193429.1
KJ747227.1	MARV	361	Partial gene	https://www.ncbi.nlm.nih.gov/nuccore/OP716851.1
KJ747228.1	MARV	348	Partial gene	https://www.ncbi.nlm.nih.gov/nuccore/KJ747211.1
KJ747229.1	MARV	372	Partial gene	https://www.ncbi.nlm.nih.gov/nuccore/FJ743670.1
KJ747230.1	MARV	290	Partial gene	https://www.ncbi.nlm.nih.gov/nuccore/FJ743670.1
KJ747231.1	MARV	289	Partial gene	https://www.ncbi.nlm.nih.gov/nuccore/JX462499.1
KJ747232.1	MARV	346	Partial gene	https://www.ncbi.nlm.nih.gov/nuccore/FJ743669.1
KJ747233.1	MARV	324	Partial gene	https://www.ncbi.nlm.nih.gov/nuccore/JX462506.1
KJ747234.1	MARV	351	Partial gene	https://www.ncbi.nlm.nih.gov/nuccore/JX462507.1
KJ747235.1	MARV	213	Partial gene	https://www.ncbi.nlm.nih.gov/nuccore/KJ747225.1
KJ747236.1	MARV	208	Partial gene	https://www.ncbi.nlm.nih.gov/nuccore/DQ466116.1
KJ747239.1	MARV	213	Partial gene	https://www.ncbi.nlm.nih.gov/nuccore/KJ747233.1
KJ747243.1	MARV	213	Partial gene	https://www.ncbi.nlm.nih.gov/nuccore/JX462505.1
KJ747244.1	MARV	207	Partial gene	https://www.ncbi.nlm.nih.gov/nuccore/EU118796.1
KJ747245.1	MARV	209	Partial gene	https://www.ncbi.nlm.nih.gov/nuccore/EU118794.1
KJ747246.1	MARV	211	Partial gene	https://www.ncbi.nlm.nih.gov/nuccore/EU118795.1
KJ747248.1	MARV	203	Partial gene	https://www.ncbi.nlm.nih.gov/nuccore/EU118799.1
KJ747249.1	MARV	208	Partial gene	https://www.ncbi.nlm.nih.gov/nuccore/DQ466112.1
KJ747250.1	MARV	203	Partial gene	https://www.ncbi.nlm.nih.gov/nuccore/DQ466108.1
KJ747251.1	MARV	208	Partial gene	https://www.ncbi.nlm.nih.gov/nuccore/DQ466135.1
KM261523.1	MARV	19114	Lab adapt isolate	https://www.ncbi.nlm.nih.gov/nuccore/EU118798.1
KP117259.1	MARV	19113	Recombinant	https://www.ncbi.nlm.nih.gov/nuccore/DQ466131.1
KP117260.1	MARV	19113	Recombinant	https://www.ncbi.nlm.nih.gov/nuccore/EU068112.1
KP117261.1	MARV	19113	Recombinant	https://www.ncbi.nlm.nih.gov/nuccore/DQ466138.1
KY047764.1	MARV	19025	Lab adapt isolate	https://www.ncbi.nlm.nih.gov/nuccore/JX462491.1
LC465155.1	MARV	563	Partial gene	https://www.ncbi.nlm.nih.gov/nuccore/FJ743679.1
LC465156.1	MARV	302	Partial gene	https://www.ncbi.nlm.nih.gov/nuccore/DQ466110.1
LC465157.1	MARV	238	Partial gene	https://www.ncbi.nlm.nih.gov/nuccore/FJ743681.1

LC465158.1	MARV	464	Partial gene	https://www.ncbi.nlm.nih.gov/nuccore/JQ272621.1
M72714.1	MARV	3000	Single gene	https://www.ncbi.nlm.nih.gov/nuccore/DQ466143.1
M92834.1	MARV	7818	Single gene	https://www.ncbi.nlm.nih.gov/nuccore/EU118804.1
MF871598.1	MARV	2088	Recombinant	https://www.ncbi.nlm.nih.gov/nuccore/EU068113.1
MF939097.1	MARV	19114	Lab adapt isolate	https://www.ncbi.nlm.nih.gov/nuccore/LC465156.1
MK271062.1	MARV	19875	Recombinant	https://www.ncbi.nlm.nih.gov/nuccore/EU068111.1
MN193419.1	MARV	411	Partial gene	https://www.ncbi.nlm.nih.gov/nuccore/FJ743683.1
MN193420.1	MARV	389	Partial gene	https://www.ncbi.nlm.nih.gov/nuccore/FJ743685.1
MN193421.1	MARV	384	Partial gene	https://www.ncbi.nlm.nih.gov/nuccore/KJ747231.1
MN193422.1	MARV	382	Partial gene	https://www.ncbi.nlm.nih.gov/nuccore/JX462503.1
MN193423.1	MARV	419	Partial gene	https://www.ncbi.nlm.nih.gov/nuccore/KJ747230.1
MN193424.1	MARV	423	Partial gene	https://www.ncbi.nlm.nih.gov/nuccore/DQ466181.1
MN193425.1	MARV	464	Partial gene	https://www.ncbi.nlm.nih.gov/nuccore/DQ466184.1
MN193426.1	MARV	464	Partial gene	https://www.ncbi.nlm.nih.gov/nuccore/DQ466175.1
MN193427.1	MARV	412	Partial gene	https://www.ncbi.nlm.nih.gov/nuccore/DQ466179.1
MN193429.1	MARV	302	Partial gene	https://www.ncbi.nlm.nih.gov/nuccore/DQ466174.1
MN193430.1	MARV	192	Partial gene	https://www.ncbi.nlm.nih.gov/nuccore/DQ466194.1
MN193431.1	MARV	162	Partial gene	https://www.ncbi.nlm.nih.gov/nuccore/DQ466195.1
MT929356.1	MARV	587	Partial gene	https://www.ncbi.nlm.nih.gov/nuccore/DQ466176.1
MT929357.1	MARV	587	Partial gene	https://www.ncbi.nlm.nih.gov/nuccore/LC465157.1
MT929358.1	MARV	587	Partial gene	https://www.ncbi.nlm.nih.gov/nuccore/KJ747243.1
OL956937.1	MARV	20240	Recombinant	https://www.ncbi.nlm.nih.gov/nuccore/KJ747249.1
OL956939.1	MARV	19478	Recombinant	https://www.ncbi.nlm.nih.gov/nuccore/KJ747250.1
OP716850.1	MARV	1792	Partial gene	https://www.ncbi.nlm.nih.gov/nuccore/KJ747235.1
OP716851.1	MARV	300	Partial gene	https://www.ncbi.nlm.nih.gov/nuccore/KJ747235.1
OP729425.1	MARV	92	Partial gene	https://www.ncbi.nlm.nih.gov/nuccore/KJ747239.1
OQ847644.1	MARV	19115	Recombinant	https://www.ncbi.nlm.nih.gov/nuccore/KJ747245.1
X64405.1	MARV	2517	Partial gene	https://www.ncbi.nlm.nih.gov/nuccore/KJ747236.1
X64406.1	MARV	2906	Partial gene	https://www.ncbi.nlm.nih.gov/nuccore/MN193430.1
X68493.1	MARV	2948	Single gene	https://www.ncbi.nlm.nih.gov/nuccore/KJ747248.1
X68494.1	MARV	7829	Single gene	https://www.ncbi.nlm.nih.gov/nuccore/KJ747251.1
X68495.1	MARV	2852	Single gene	https://www.ncbi.nlm.nih.gov/nuccore/KJ747244.1
Z12132.1	MARV	19104	Lab adapt isolate	https://www.ncbi.nlm.nih.gov/nuccore/MN193431.1
Z29337.1	MARV	19112	Lab adapt isolate	https://www.ncbi.nlm.nih.gov/nuccore/OP729425.1

Table S2: Reference sequences of MARV and RAVV genomes included in the study

Accession No.	Country of isolation	Host of isolation	Year of isolation	Marburg virus genus	Reference link
DQ447649.1	Kenya	Human	1987	RAVV	https://www.ncbi.nlm.nih.gov/nuccore/DQ447649.1
DQ447652.1	Democratic Republic of the Congo	Human	1999	RAVV	https://www.ncbi.nlm.nih.gov/nuccore/DQ447652.1
EU500827.1	Kenya	Human	1987	RAVV	https://www.ncbi.nlm.nih.gov/nuccore/EU500827.1
FJ750953.1	Uganda	Human	2007	RAVV	https://www.ncbi.nlm.nih.gov/nuccore/FJ750953.1
FJ750954.1	Uganda	Bat	2007	RAVV	https://www.ncbi.nlm.nih.gov/nuccore/FJ750954.1
FJ750955.1	Uganda	Bat	2007	RAVV	https://www.ncbi.nlm.nih.gov/nuccore/FJ750955.1
FJ750956.1	Uganda	Bat	2008	RAVV	https://www.ncbi.nlm.nih.gov/nuccore/FJ750956.1
JX458857.1	Uganda	Bat	2009	RAVV	https://www.ncbi.nlm.nih.gov/nuccore/JX458857.1
KU059750.1	Uganda	Human	2007	RAVV	https://www.ncbi.nlm.nih.gov/nuccore/KU059750.1
KU179482.1	Kenya	Human	1987	RAVV	https://www.ncbi.nlm.nih.gov/nuccore/KU179482.1
MT321489.1	South Africa	Bat	2017	RAVV	https://www.ncbi.nlm.nih.gov/nuccore/MT321489.1
NC_024781.1	Kenya	Human	1987	RAVV	https://www.ncbi.nlm.nih.gov/nuccore/NC_024781.1
AY358025.2	South Africa	Human	1975	MARV	https://www.ncbi.nlm.nih.gov/nuccore/AY358025.2
DQ447650.1	Democratic Republic of the Congo	Human	1999	MARV	https://www.ncbi.nlm.nih.gov/nuccore/DQ447650.1
DQ447651.1	Democratic Republic of the Congo	Human	1999	MARV	https://www.ncbi.nlm.nih.gov/nuccore/DQ447651.1
DQ447653.1	Angola	Human	2005	MARV	https://www.ncbi.nlm.nih.gov/nuccore/DQ447653.1
DQ447654.1	Angola	Human	2005	MARV	https://www.ncbi.nlm.nih.gov/nuccore/DQ447654.1
DQ447655.1	Angola	Human	2005	MARV	https://www.ncbi.nlm.nih.gov/nuccore/DQ447655.1
DQ447656.1	Angola	Human	2005	MARV	https://www.ncbi.nlm.nih.gov/nuccore/DQ447656.1
DQ447657.1	Angola	Human	2005	MARV	https://www.ncbi.nlm.nih.gov/nuccore/DQ447657.1
DQ447658.1	Angola	Human	2005	MARV	https://www.ncbi.nlm.nih.gov/nuccore/DQ447658.1
DQ447659.1	Angola	Human	2005	MARV	https://www.ncbi.nlm.nih.gov/nuccore/DQ447659.1
DQ447660.1	Angola	Human	2005	MARV	https://www.ncbi.nlm.nih.gov/nuccore/DQ447660.1
FJ750957.1	Uganda	Human	2007	MARV	https://www.ncbi.nlm.nih.gov/nuccore/FJ750957.1
FJ750958.1	Uganda	Bat	2007	MARV	https://www.ncbi.nlm.nih.gov/nuccore/FJ750958.1
FJ750959.1	Uganda	Bat	2007	MARV	https://www.ncbi.nlm.nih.gov/nuccore/FJ750959.1
JN408064.1	Netherlands	Human	2008	MARV	https://www.ncbi.nlm.nih.gov/nuccore/JN408064.1
JX458825.1	Democratic Republic of the Congo	Human	1999	MARV	https://www.ncbi.nlm.nih.gov/nuccore/JX458825.1
JX458826.1	Democratic Republic of the Congo	Human	1999	MARV	https://www.ncbi.nlm.nih.gov/nuccore/JX458826.1
JX458827.1	Democratic Republic of the Congo	Human	2000	MARV	https://www.ncbi.nlm.nih.gov/nuccore/JX458827.1
JX458828.1	Democratic Republic of the Congo	Human	2000	MARV	https://www.ncbi.nlm.nih.gov/nuccore/JX458828.1
JX458829.1	Democratic Republic of the Congo	Human	2000	MARV	https://www.ncbi.nlm.nih.gov/nuccore/JX458829.1

JX458830.1	Democratic Republic of the Congo	Human	1999	MARV	https://www.ncbi.nlm.nih.gov/nuccore/JX458830.1
JX458831.1	Democratic Republic of the Congo	Human	2000	MARV	https://www.ncbi.nlm.nih.gov/nuccore/JX458831.1
JX458832.1	Democratic Republic of the Congo	Human	2000	MARV	https://www.ncbi.nlm.nih.gov/nuccore/JX458832.1
JX458833.1	Democratic Republic of the Congo	Human	1999	MARV	https://www.ncbi.nlm.nih.gov/nuccore/JX458833.1
JX458834.1	Democratic Republic of the Congo	Human	2000	MARV	https://www.ncbi.nlm.nih.gov/nuccore/JX458834.1
JX458835.1	Democratic Republic of the Congo	Human	2000	MARV	https://www.ncbi.nlm.nih.gov/nuccore/JX458835.1
JX458836.1	Democratic Republic of the Congo	Human	2000	MARV	https://www.ncbi.nlm.nih.gov/nuccore/JX458836.1
JX458837.1	Democratic Republic of the Congo	Human	2000	MARV	https://www.ncbi.nlm.nih.gov/nuccore/JX458837.1
JX458838.1	Democratic Republic of the Congo	Human	2000	MARV	https://www.ncbi.nlm.nih.gov/nuccore/JX458838.1
JX458839.1	Democratic Republic of the Congo	Human	2000	MARV	https://www.ncbi.nlm.nih.gov/nuccore/JX458839.1
JX458840.1	Democratic Republic of the Congo	Human	2000	MARV	https://www.ncbi.nlm.nih.gov/nuccore/JX458840.1
JX458841.1	Democratic Republic of the Congo	Human	2000	MARV	https://www.ncbi.nlm.nih.gov/nuccore/JX458841.1
JX458842.1	Democratic Republic of the Congo	Human	2000	MARV	https://www.ncbi.nlm.nih.gov/nuccore/JX458842.1
JX458843.1	Democratic Republic of the Congo	Human	2000	MARV	https://www.ncbi.nlm.nih.gov/nuccore/JX458843.1
JX458844.1	Democratic Republic of the Congo	Human	2000	MARV	https://www.ncbi.nlm.nih.gov/nuccore/JX458844.1
JX458845.1	Democratic Republic of the Congo	Human	2000	MARV	https://www.ncbi.nlm.nih.gov/nuccore/JX458845.1
JX458846.1	Democratic Republic of the Congo	Human	2000	MARV	https://www.ncbi.nlm.nih.gov/nuccore/JX458846.1
JX458847.1	Democratic Republic of the Congo	Human	2000	MARV	https://www.ncbi.nlm.nih.gov/nuccore/JX458847.1
JX458848.1	Democratic Republic of the Congo	Human	2000	MARV	https://www.ncbi.nlm.nih.gov/nuccore/JX458848.1
JX458849.1	Democratic Republic of the Congo	Human	2000	MARV	https://www.ncbi.nlm.nih.gov/nuccore/JX458849.1
JX458850.1	Democratic Republic of the Congo	Human	2000	MARV	https://www.ncbi.nlm.nih.gov/nuccore/JX458850.1
JX458851.1	Democratic Republic of the Congo	Human	1999	MARV	https://www.ncbi.nlm.nih.gov/nuccore/JX458851.1
JX458852.1	Uganda	Bat	2008	MARV	https://www.ncbi.nlm.nih.gov/nuccore/JX458852.1
JX458853.1	Uganda	Bat	2008	MARV	https://www.ncbi.nlm.nih.gov/nuccore/JX458853.1
JX458854.1	Uganda	Bat	2009	MARV	https://www.ncbi.nlm.nih.gov/nuccore/JX458854.1
JX458855.1	Uganda	Bat	2009	MARV	https://www.ncbi.nlm.nih.gov/nuccore/JX458855.1
JX458856.1	Uganda	Bat	2009	MARV	https://www.ncbi.nlm.nih.gov/nuccore/JX458856.1
JX458858.1	Uganda	Bat	2009	MARV	https://www.ncbi.nlm.nih.gov/nuccore/JX458858.1
KC545387.1	Uganda	Human	2012	MARV	https://www.ncbi.nlm.nih.gov/nuccore/KC545387.1
KC545388.1	Uganda	Human	2012	MARV	https://www.ncbi.nlm.nih.gov/nuccore/KC545388.1
KP985768.1	Uganda	Human	2014	MARV	https://www.ncbi.nlm.nih.gov/nuccore/KP985768.1
KR063674.1	Angola	Human	2005	MARV	https://www.ncbi.nlm.nih.gov/nuccore/KR063674.1
KU978782.1	Angola	Human	2005	MARV	https://www.ncbi.nlm.nih.gov/nuccore/KU978782.1
KY047763.1	Angola	Human	2005	MARV	https://www.ncbi.nlm.nih.gov/nuccore/KY047763.1
KY425629.1	Angola	Human	2005	MARV	https://www.ncbi.nlm.nih.gov/nuccore/KY425629.1
MG725616.1	South Africa	Bat	2013	MARV	https://www.ncbi.nlm.nih.gov/nuccore/MG725616.1

MH638314.1	Uganda	Human	2017	MARV	https://www.ncbi.nlm.nih.gov/nuccore/MH638314.1
MH638315.1	Uganda	Human	2017	MARV	https://www.ncbi.nlm.nih.gov/nuccore/MH638315.1
MN187403.1	Sierra Leone	Bat	2018	MARV	https://www.ncbi.nlm.nih.gov/nuccore/MN187403.1
MN187404.1	Sierra Leone	Bat	2018	MARV	https://www.ncbi.nlm.nih.gov/nuccore/MN187404.1
MN187405.1	Sierra Leone	Bat	2018	MARV	https://www.ncbi.nlm.nih.gov/nuccore/MN187405.1
MN187406.1	Sierra Leone	Bat	2018	MARV	https://www.ncbi.nlm.nih.gov/nuccore/MN187406.1
MN258361.1	Sierra Leone	Bat	2017	MARV	https://www.ncbi.nlm.nih.gov/nuccore/MN258361.1
MN258362.1	Sierra Leone	Bat	2017	MARV	https://www.ncbi.nlm.nih.gov/nuccore/MN258362.1
MT586762.1	Angola	Human	2005	MARV	https://www.ncbi.nlm.nih.gov/nuccore/MT586762.1
NC_001608.3	Kenya	Human	1980	MARV	https://www.ncbi.nlm.nih.gov/nuccore/NC_001608.3
OK665848.1	Guinea	Human	2021	MARV	https://www.ncbi.nlm.nih.gov/nuccore/OK665848.1
OL702894.1	Guinea	Human	2021	MARV	https://www.ncbi.nlm.nih.gov/nuccore/OL702894.1
OQ672470.1	Ghana	Human	2022	MARV	https://www.ncbi.nlm.nih.gov/nuccore/OQ672470.1
OQ672471.1	Ghana	Human	2022	MARV	https://www.ncbi.nlm.nih.gov/nuccore/OQ672471.1

Table S3: Reference sequences of genomes included in the study as outgroup taxa

Accession No.	Virus genus	Reference link
AB050936.1	*Reston ebolavirus*	https://www.ncbi.nlm.nih.gov/nuccore/AB050936.1
JN638998.1	*Sudan ebolavirus*	https://www.ncbi.nlm.nih.gov/nuccore/JN638998.1
KU182898.1	*Ebola virus*	https://www.ncbi.nlm.nih.gov/nuccore/KU182898.1
LT605058.1	*Ebola virus*	https://www.ncbi.nlm.nih.gov/nuccore/LT605058.1
NC_014373.1	*Bundibugyo ebolavirus*	https://www.ncbi.nlm.nih.gov/nuccore/NC_014373.1
NC_016144.1	*Lloviu cuevavirus*	https://www.ncbi.nlm.nih.gov/nuccore/NC_016144.1
NC_039345.1	*Bombali ebolavirus*	https://www.ncbi.nlm.nih.gov/nuccore/NC_039345.1
NC_055175.1	*Wenling frogfish filovirus*	https://www.ncbi.nlm.nih.gov/nuccore/NC_055175.1
NC_055176.1	*Wenling thamnaconus septentrionalis filovirus*	https://www.ncbi.nlm.nih.gov/nuccore/NC_055176.1
NC_055510.1	*Mengla dianlovirus*	https://www.ncbi.nlm.nih.gov/nuccore/NC_055510.1
NC_076535.1	*Tapajos virus*	https://www.ncbi.nlm.nih.gov/nuccore/NC_076535.1
NC_076734.1	*Fiwi virus*	https://www.ncbi.nlm.nih.gov/nuccore/NC_076734.1
NC_076735.1	*Oberland virus*	https://www.ncbi.nlm.nih.gov/nuccore/NC_076735.1
NC_076916.1	*Kander virus*	https://www.ncbi.nlm.nih.gov/nuccore/NC_076916.1
