# Supplementary material for: Genomic Evolution and Phylodynamics of the Species Orthomarburgvirus marburgense (Marburg and Ravn Viruses) to Understand Viral Adaptation and Marburg Virus Disease’s Transmission Dynamics
Source: Pathogens. 2024 Dec 14;13(12):1107. doi: 10.3390/pathogens13121107 (PMC11728648; doi:10.3390/pathogens13121107)
Supplement: Supplementary file 1 [file pathogens-13-01107-s001.zip › pathogens-3347639-supplementary/Supplementary File S2.pdf]

Supplementary File 2:

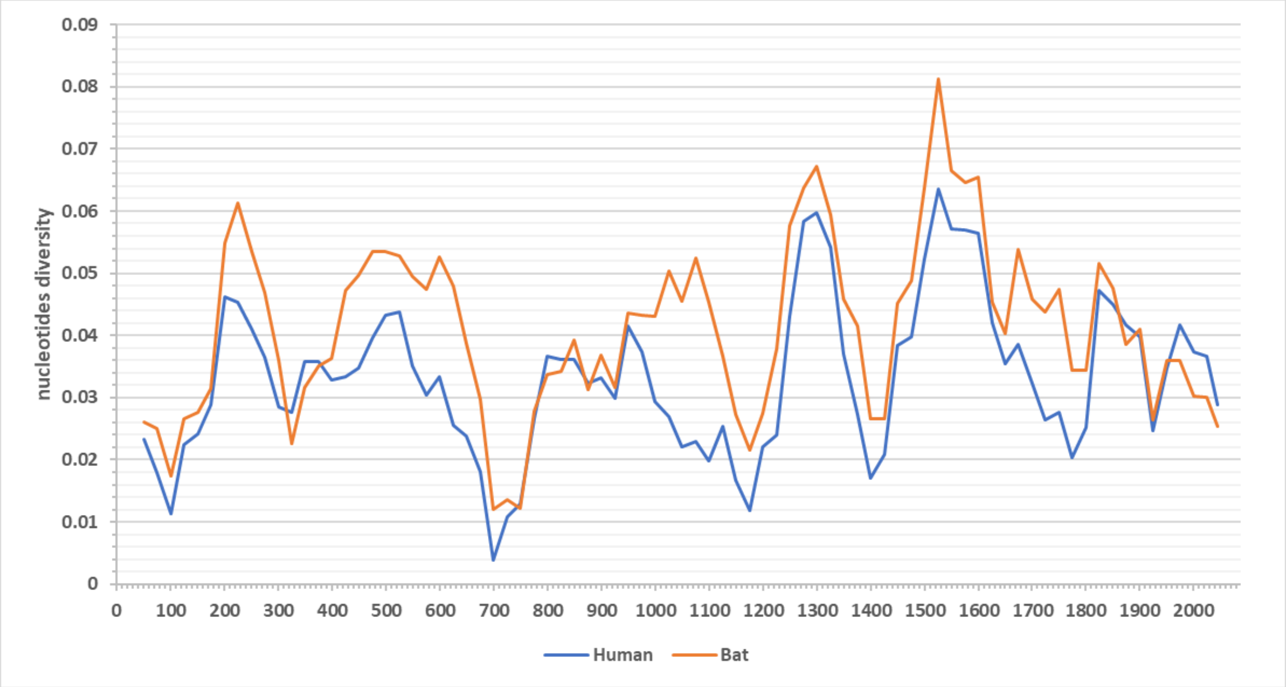

Figure S1: Sliding window analysis of the NP (Nucleoprotein) gene sequences. Blue line represents nucleotides diversity of MARV protein coding sequences of the NP gene isolated from human. Orange line represents nucleotides diversity of MARV protein coding sequences of the NP gene isolated from bats.

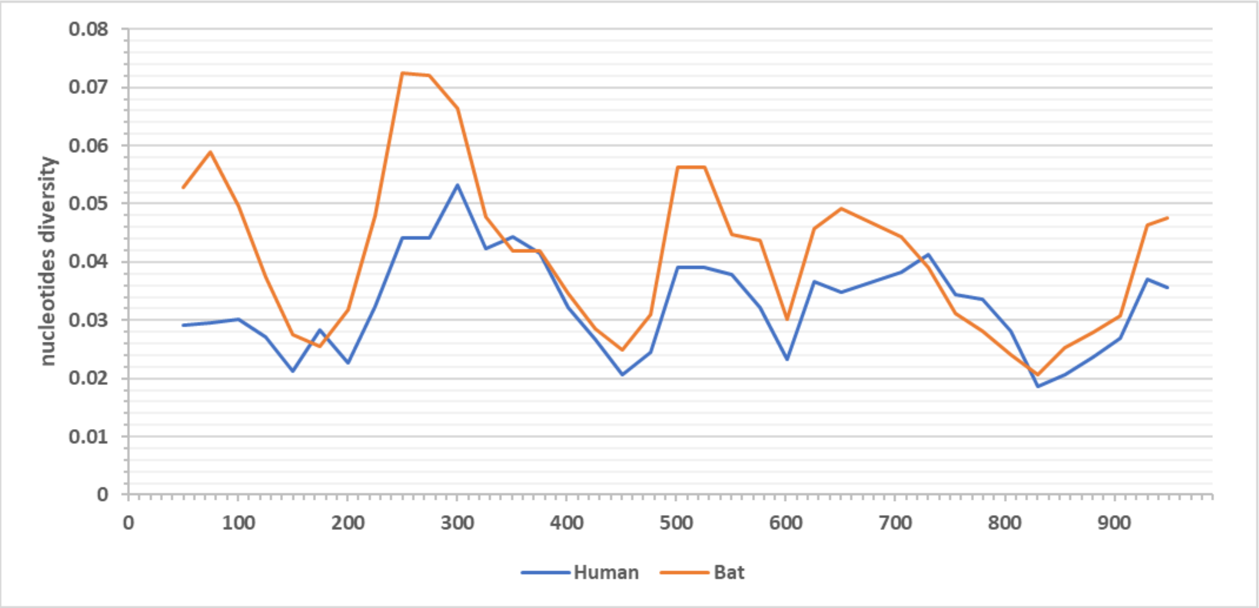

Figure S2: Sliding window analysis of the VP35 (Viral Protein 35) gene sequences. Blue line represents nucleotides diversity of MARV protein coding sequences of the VP35 gene isolated from human. Orange line represents nucleotides diversity of MARV protein coding sequences of the VP35 gene isolated from bats.

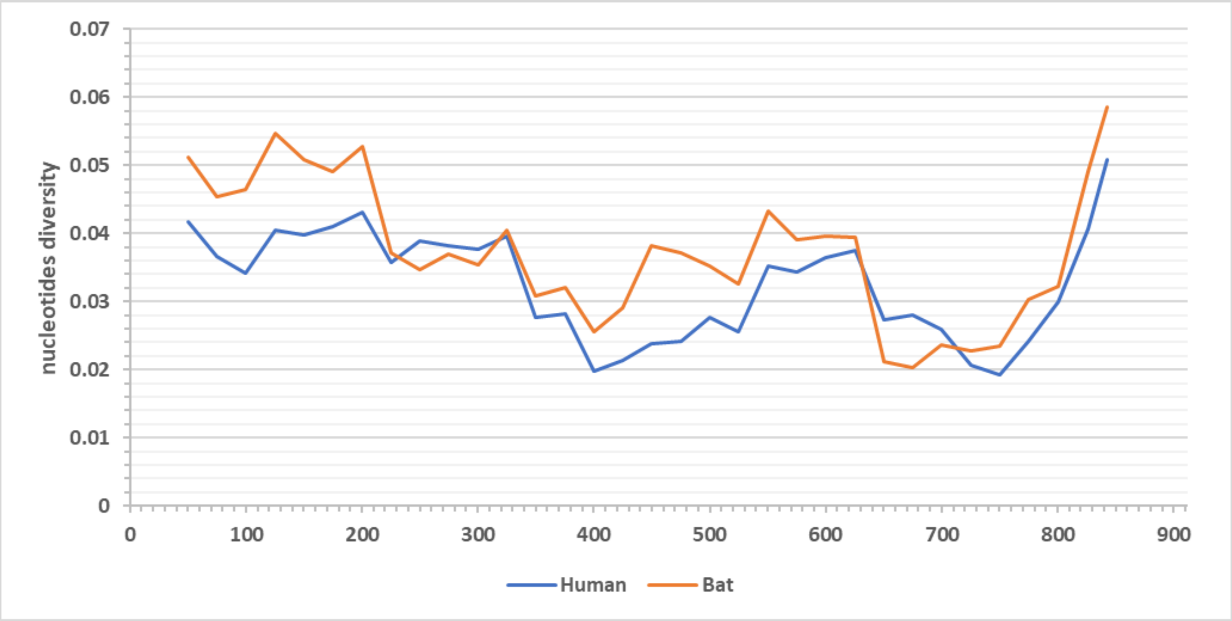

Figure S3: Sliding window analysis of the VP40 (Viral Protein 40) gene sequences. Blue line represents nucleotides diversity of MARV protein coding sequences of the VP40 gene isolated from human. Orange line represents nucleotides diversity of MARV protein coding sequences of the VP40 gene isolated from bats.

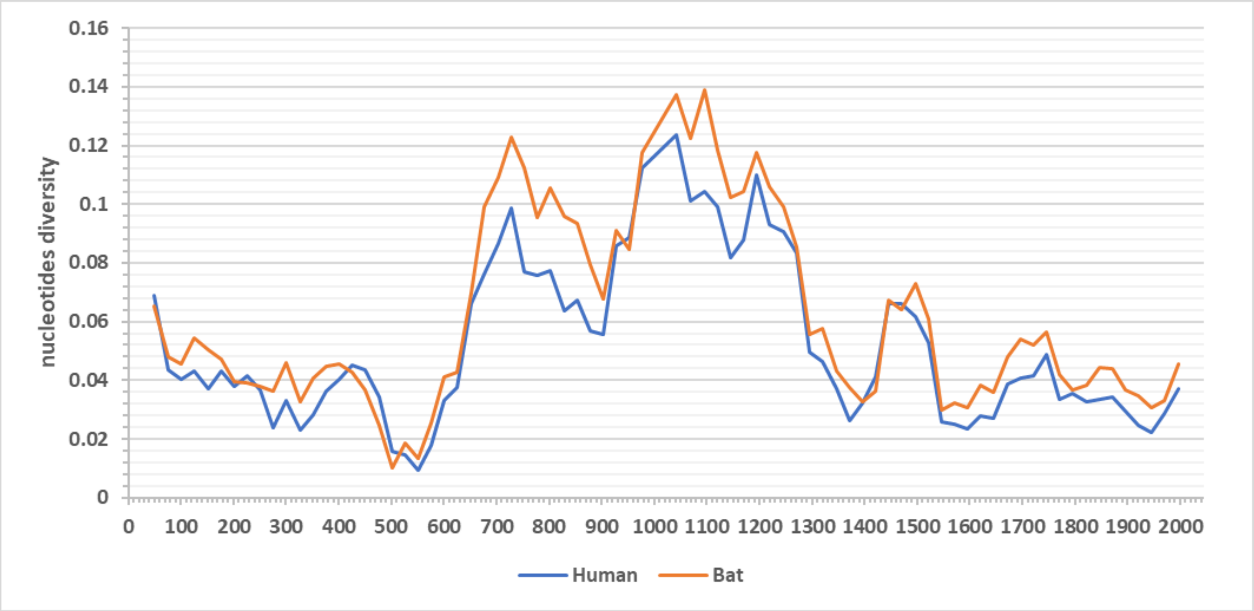

Figure S4: Sliding window analysis of the GP (Glycoprotein) gene sequences. Blue line represents nucleotides diversity of MARV protein coding sequences of the GP gene isolated from human. Orange line represents nucleotides diversity of MARV protein coding sequences of the GP gene isolated from bats.

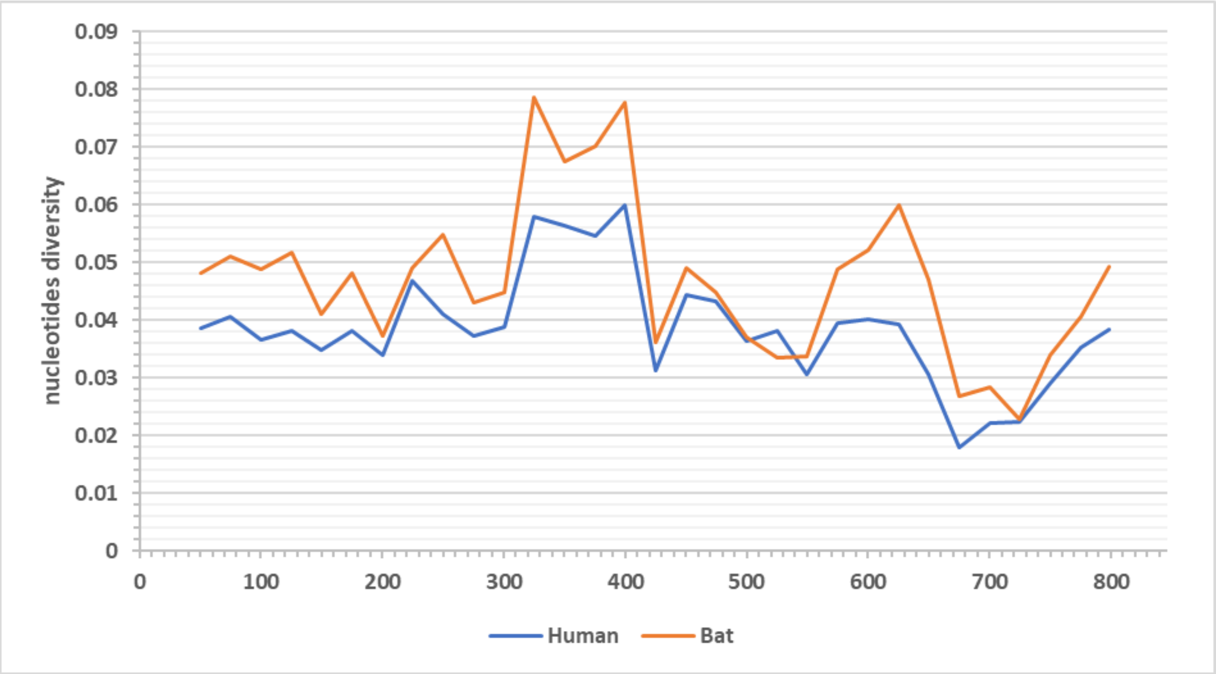

Figure S5: Sliding window analysis of the VP30 (Viral Protein 30) gene sequences. Blue line represents nucleotides diversity of MARV protein coding sequences of the VP30 gene isolated from human. Orange line represents nucleotides diversity of MARV protein coding sequences of the VP30 gene isolated from bats.

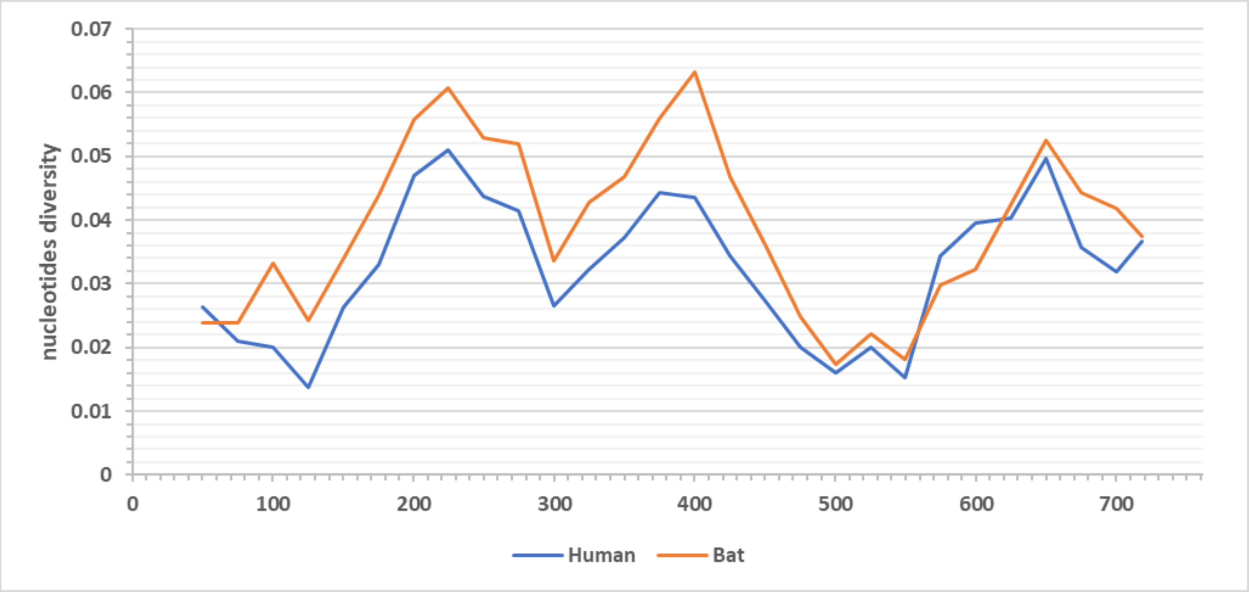

Figure S6: Sliding window analysis of the VP24 (Viral Protein 24) gene sequences. Blue line represents nucleotides diversity of MARV protein coding sequences of the VP24 gene isolated from human. Orange line represents nucleotides diversity of MARV protein coding sequences of the VP24 gene isolated from bats.

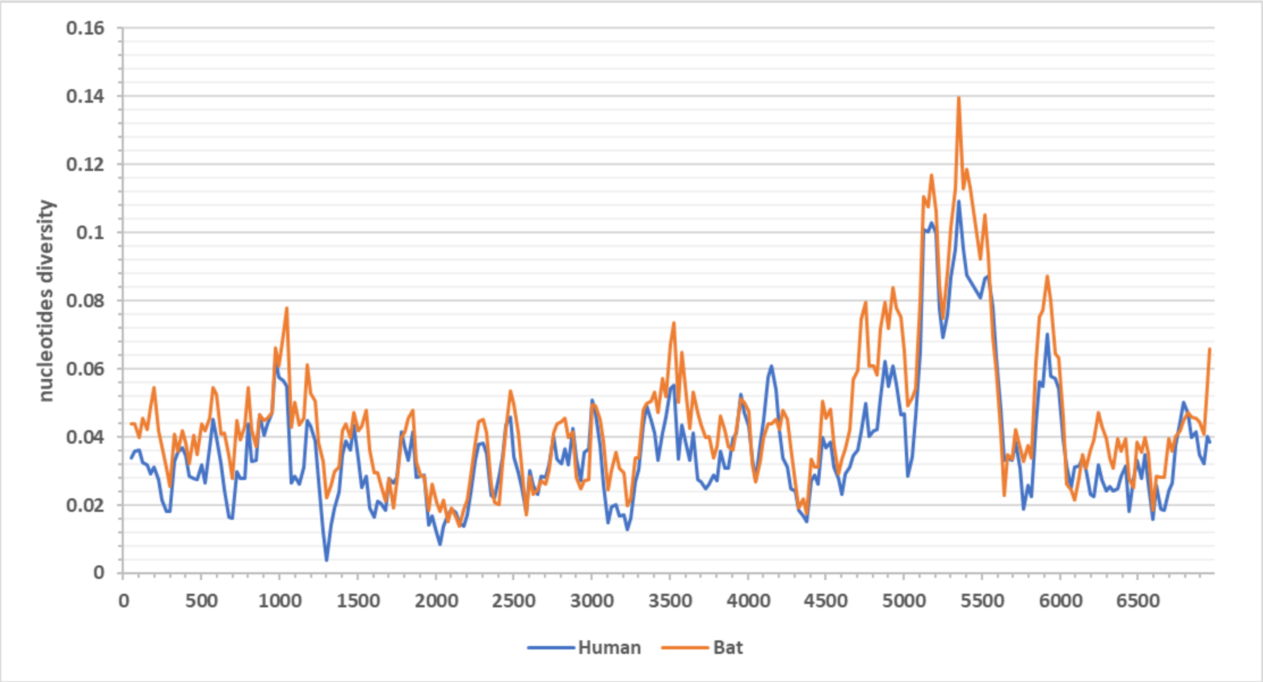

Figure S7: Sliding window analysis of the L (RNA-dependent RNA polymerase) gene sequences. Blue line represents nucleotides diversity of MARV protein coding sequences of the L gene isolated from human. Orange line represents nucleotides diversity of MARV protein coding sequences of the L gene isolated from bats.

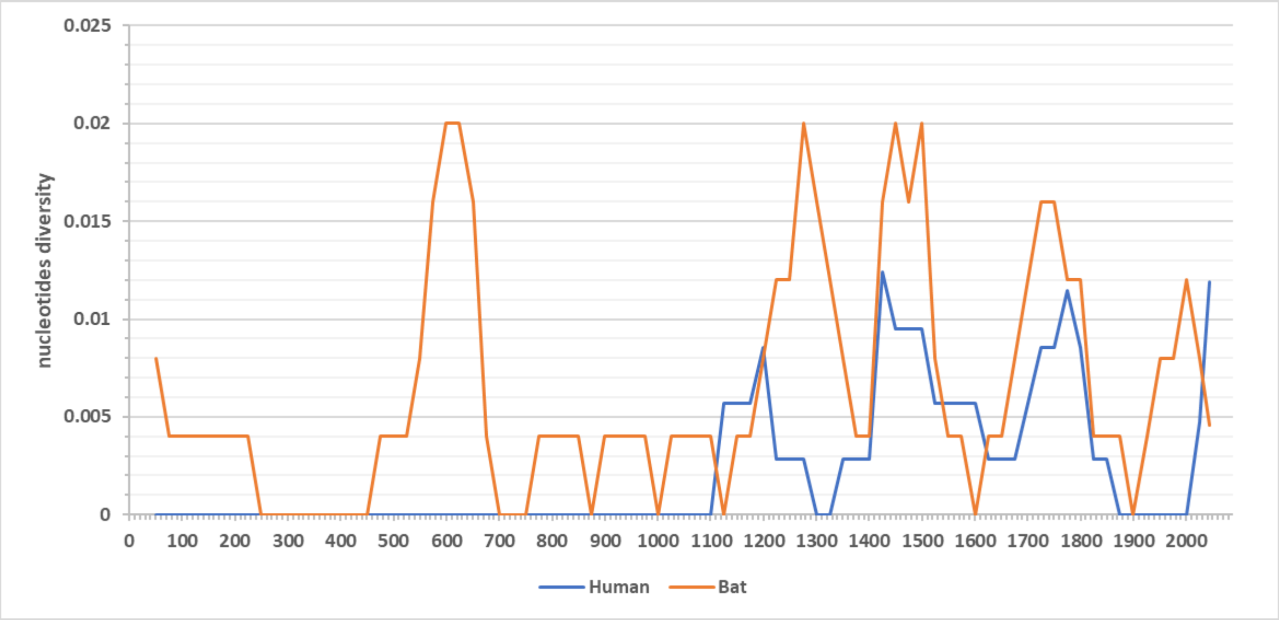

Figure S8: Sliding window analysis of the NP (Nucleoprotein) gene sequences. Blue line represents nucleotides diversity of RAVV protein coding sequences of the NP gene isolated from human. Orange line represents nucleotides diversity of RAVV protein coding sequences of the NP gene isolated from bats.

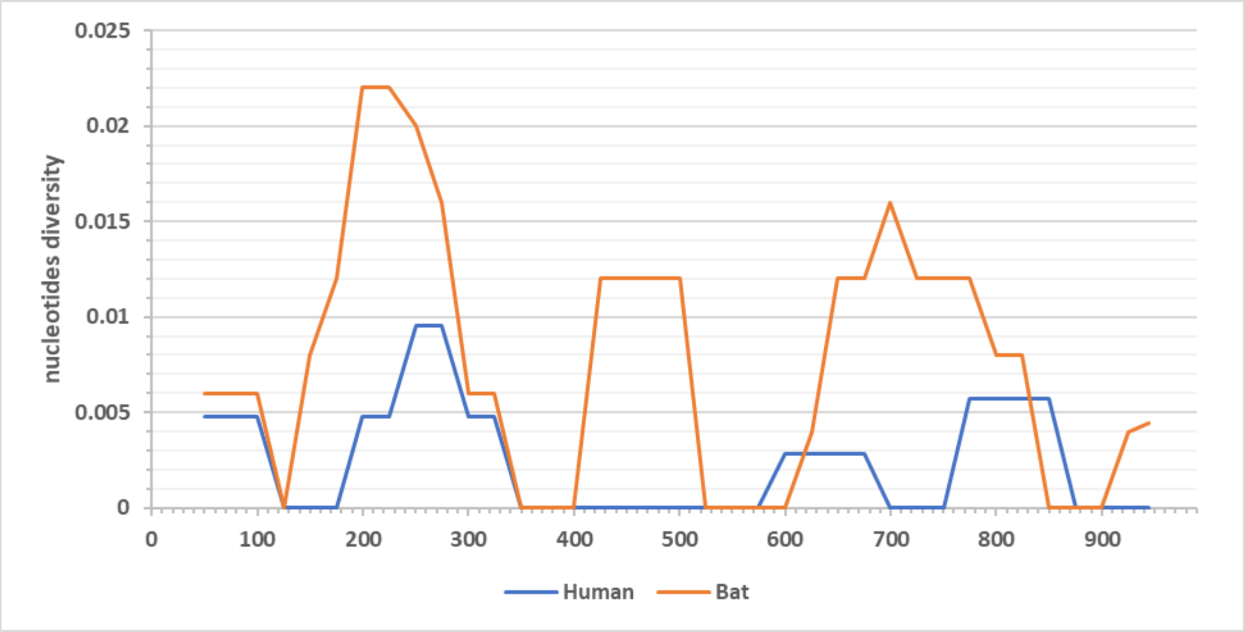

Figure S9: Sliding window analysis of the VP35 (Viral Protein 35) gene sequences. Blue line represents nucleotides diversity of RAVV protein coding sequences of the VP35 gene isolated from human. Orange line represents nucleotides diversity of RAVV protein coding sequences of the VP35 gene isolated from bats.

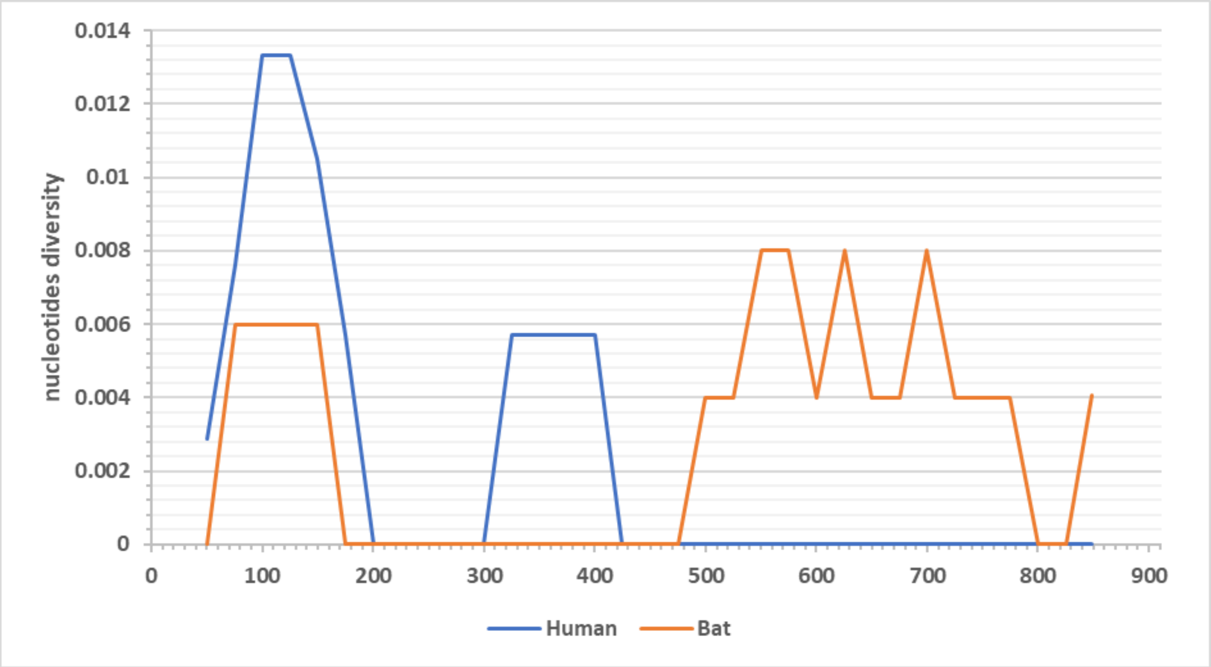

Figure S10: Sliding window analysis of the VP40 (Viral Protein 40) gene sequences. Blue line represents nucleotides diversity of RAVV protein coding sequences of the VP40 gene isolated from human. Orange line represents nucleotides diversity of RAVV protein coding sequences of the VP40 gene isolated from bats.

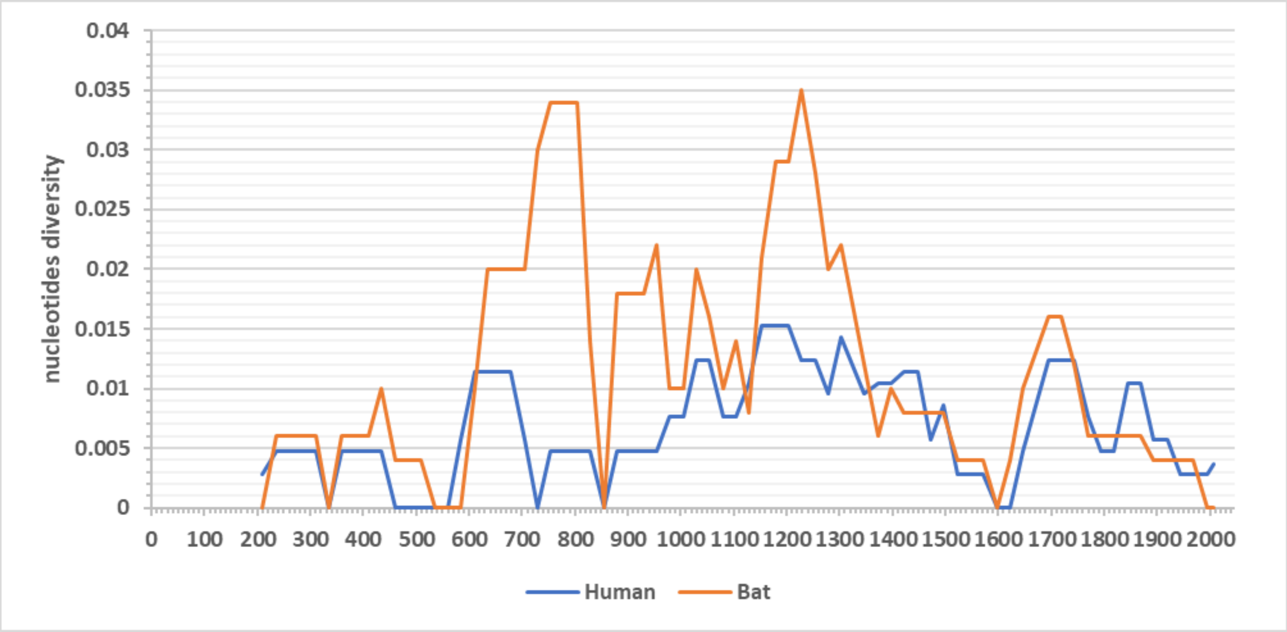

Figure S11: Sliding window analysis of the GP (Glycoprotein) gene sequences. Blue line represents nucleotides diversity of RAVV protein coding sequences of the GP gene isolated from human. Orange line represents nucleotides diversity of RAVV protein coding sequences of the GP gene isolated from bats.

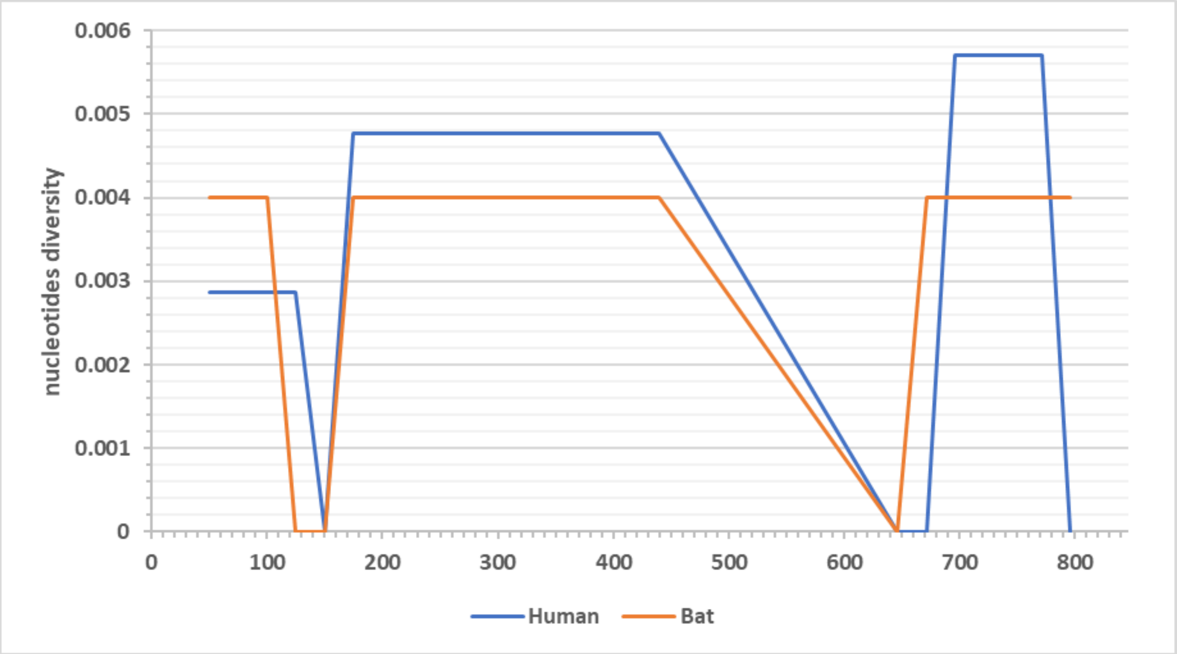

Figure S12: Sliding window analysis of the VP30 (Viral Protein 30) gene sequences. Blue line represents nucleotides diversity of RAVV protein coding sequences of the VP30 gene isolated from human. Orange line represents nucleotides diversity of RAVV protein coding sequences of the VP30 gene isolated from bats.

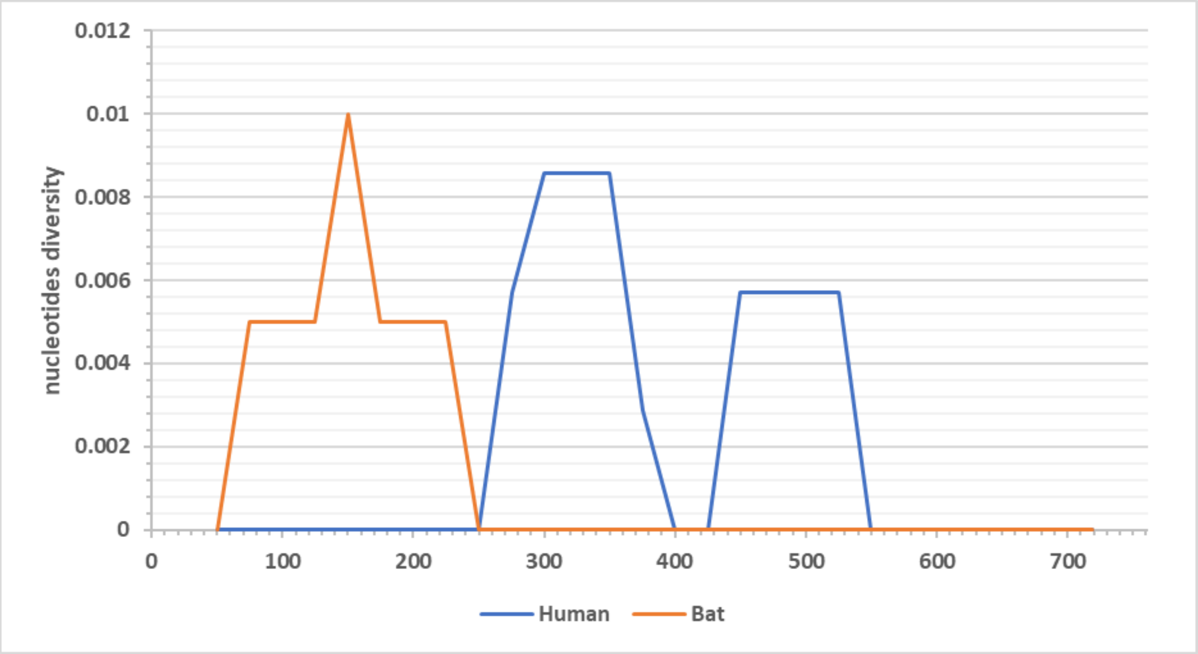

Figure S13: Sliding window analysis of the VP24 (Viral Protein 24) gene sequences. Blue line represents nucleotides diversity of RAVV protein coding sequences of the VP24 gene isolated from human. Orange line represents nucleotides diversity of RAVV protein coding sequences of the VP24 gene isolated from bats.

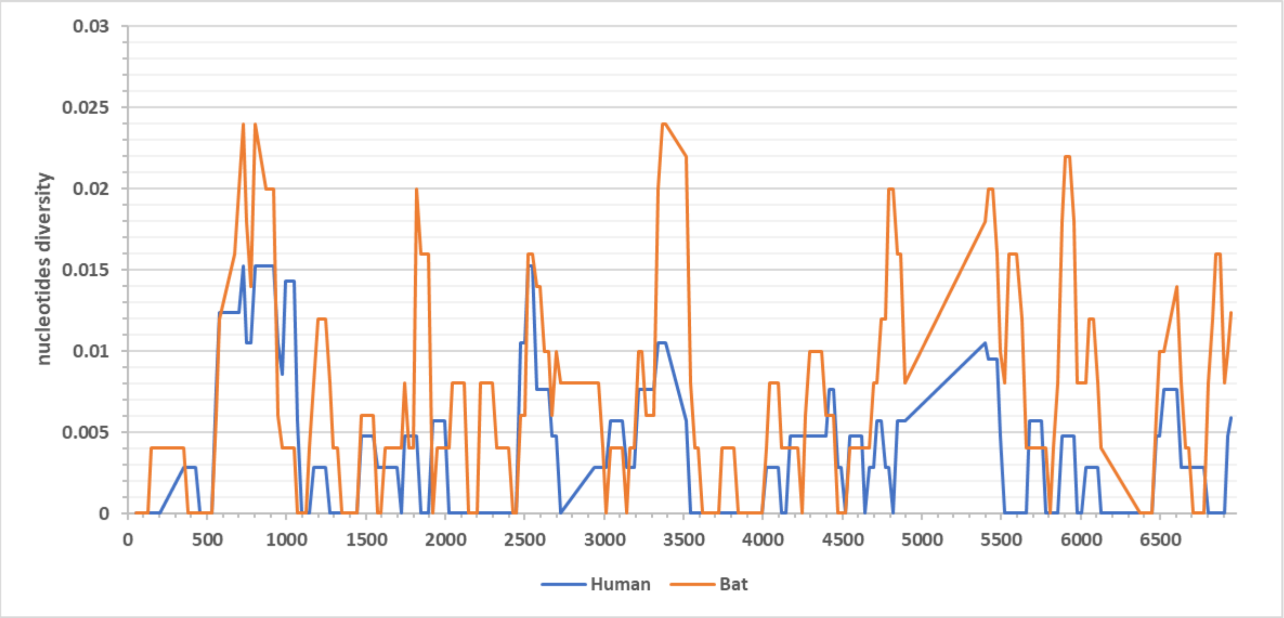

Figure S14: Sliding window analysis of the L (RNA-dependent RNA polymerase) gene sequences. Blue line represents nucleotides diversity of RAVV protein coding sequences of the L gene isolated from human. Orange line represents nucleotides diversity of RAVV protein coding sequences of the L gene isolated from bats.

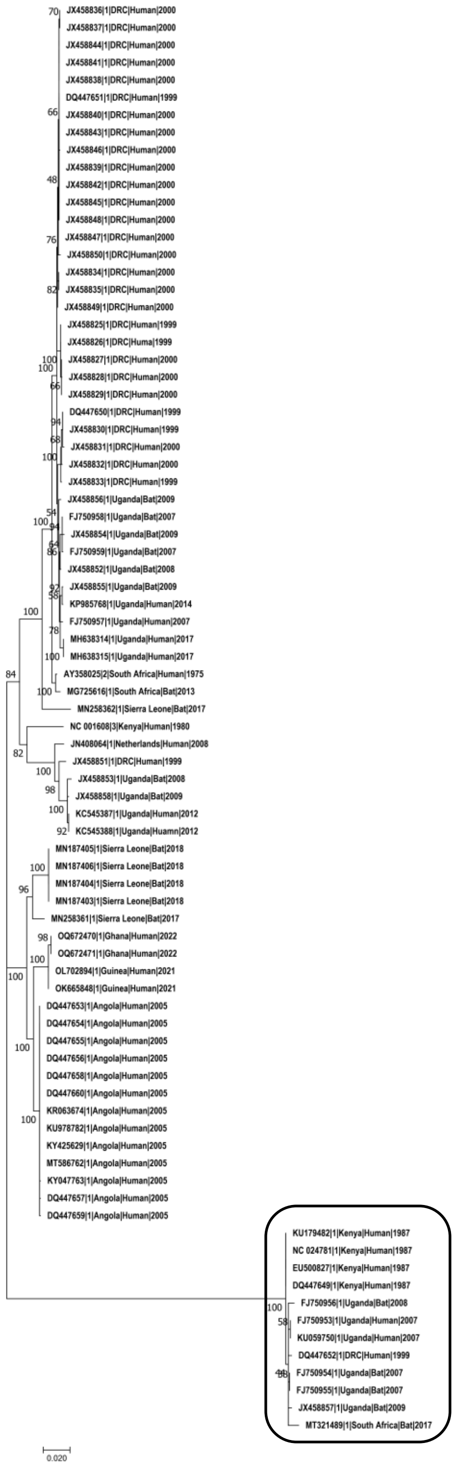

Figure S15: Maximum-Likelihood Phylogenetic Tree. The phylogenetic tree of MARV and RAVV NP gene sequences alongside. The black box indicates RAVV sequences. Bootstrap values supporting the clustering of branches are indicated next to the corresponding branches.

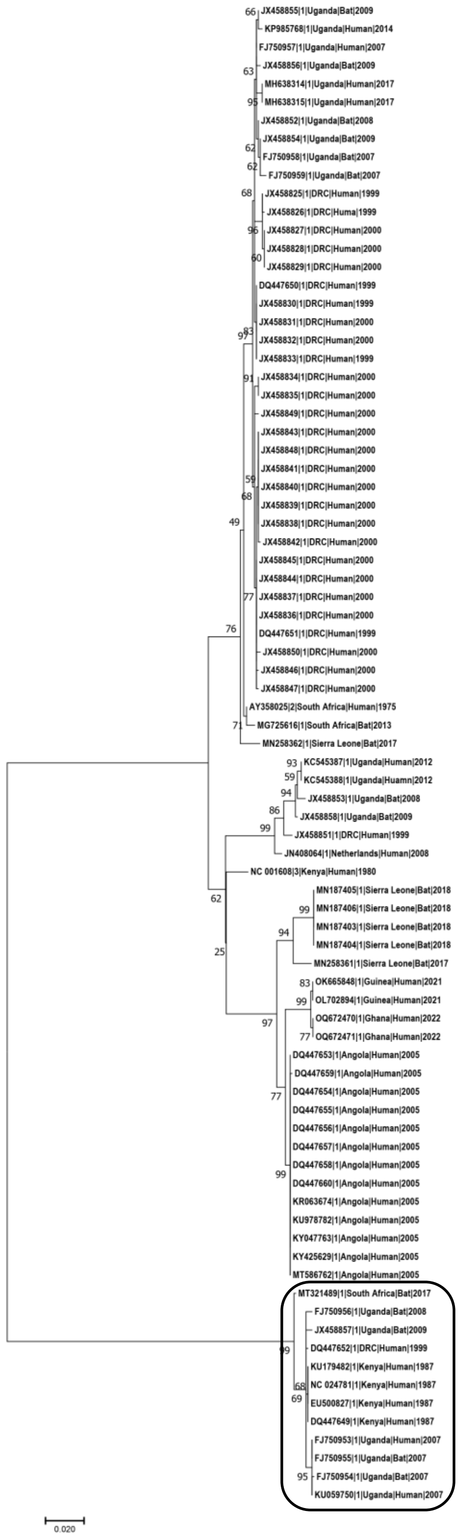

Figure S16: Maximum-Likelihood Phylogenetic Tree. The phylogenetic tree of MARV and RAVV VP35 gene sequences alongside. The black box indicates RAVV sequences. Bootstrap values supporting the clustering of branches are indicated next to the corresponding branches.

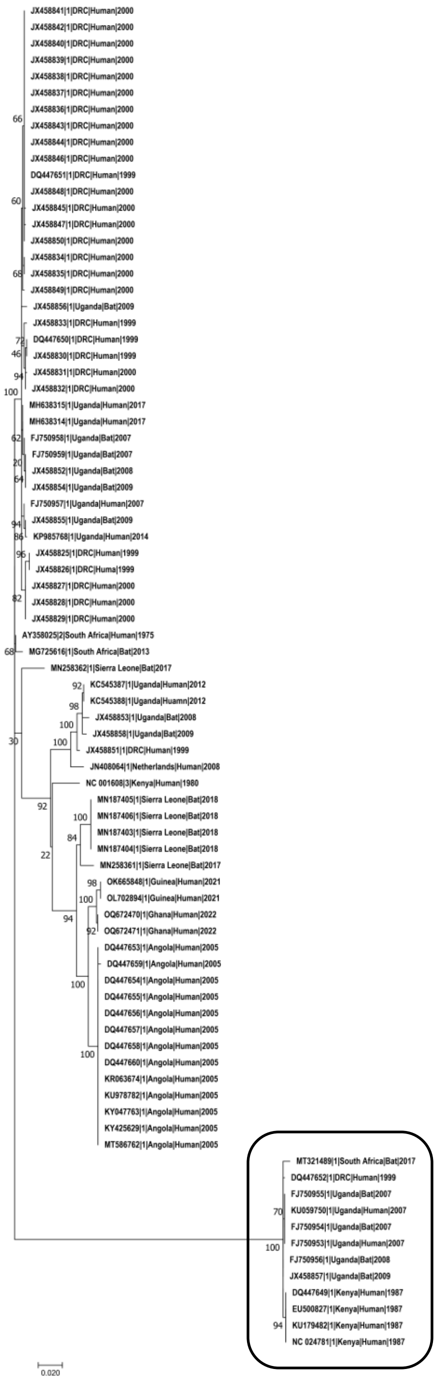

Figure S17: Maximum-Likelihood Phylogenetic Tree. The phylogenetic tree of MARV and RAVV VP40 gene sequences alongside. The black box indicates RAVV sequences. Bootstrap values supporting the clustering of branches are indicated next to the corresponding branches.

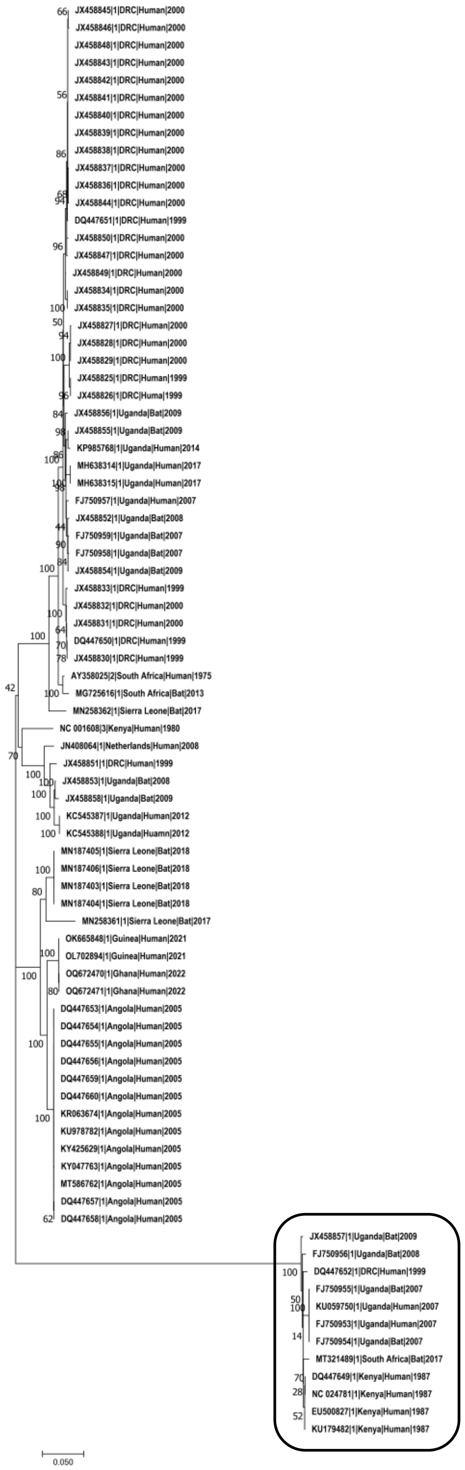

Figure S18: Maximum-Likelihood Phylogenetic Tree. The phylogenetic tree of MARV and RAVV GP gene sequences alongside. The black box indicates RAVV sequences. Bootstrap values supporting the clustering of branches are indicated next to the corresponding branches.

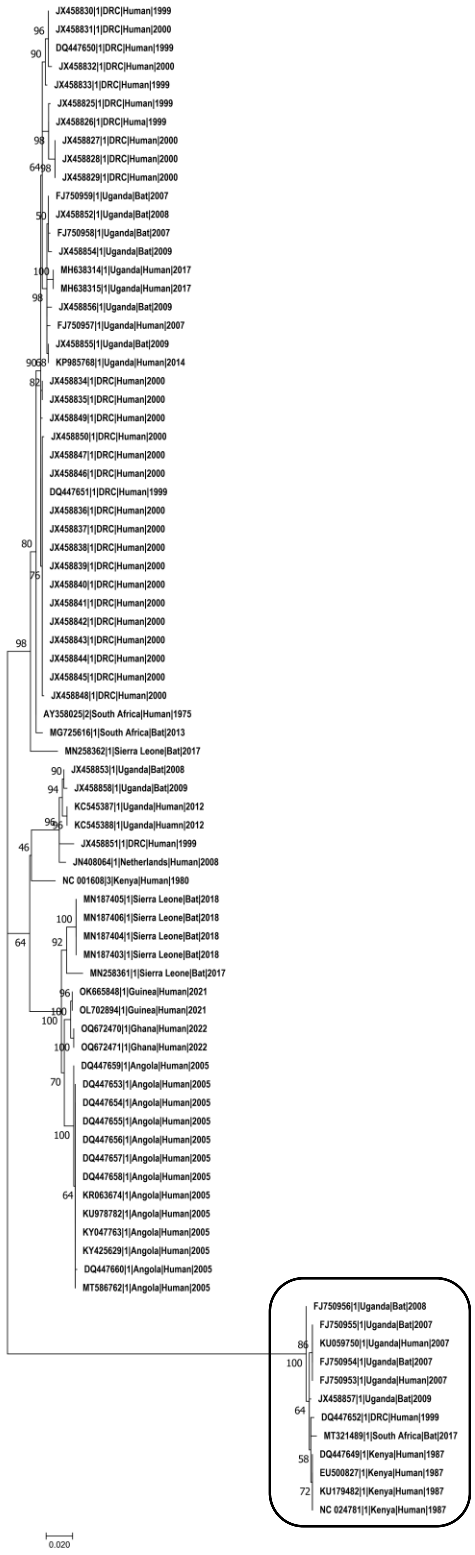

Figure S19: Maximum-Likelihood Phylogenetic Tree. The phylogenetic tree of MARV and RAVV VP30 gene sequences alongside. The black box indicates RAVV sequences. Bootstrap values supporting the clustering of branches are indicated next to the corresponding branches.

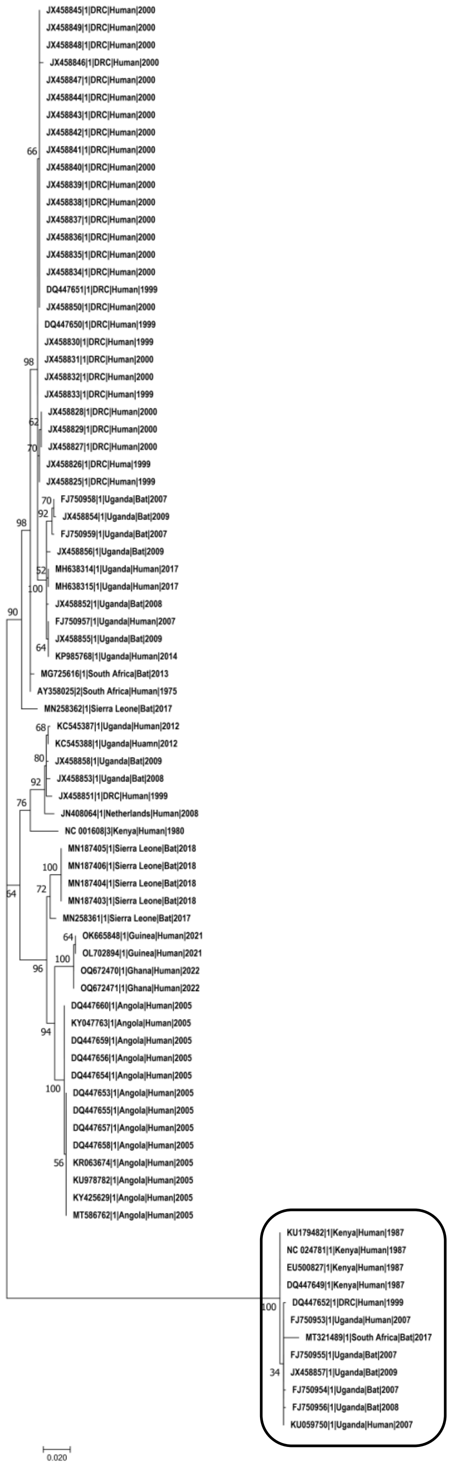

Figure S20: Maximum-Likelihood Phylogenetic Tree. The phylogenetic tree of MARV and RAVV VP24 gene sequences alongside. The black box indicates RAVV sequences. Bootstrap values supporting the clustering of branches are indicated next to the corresponding branches.

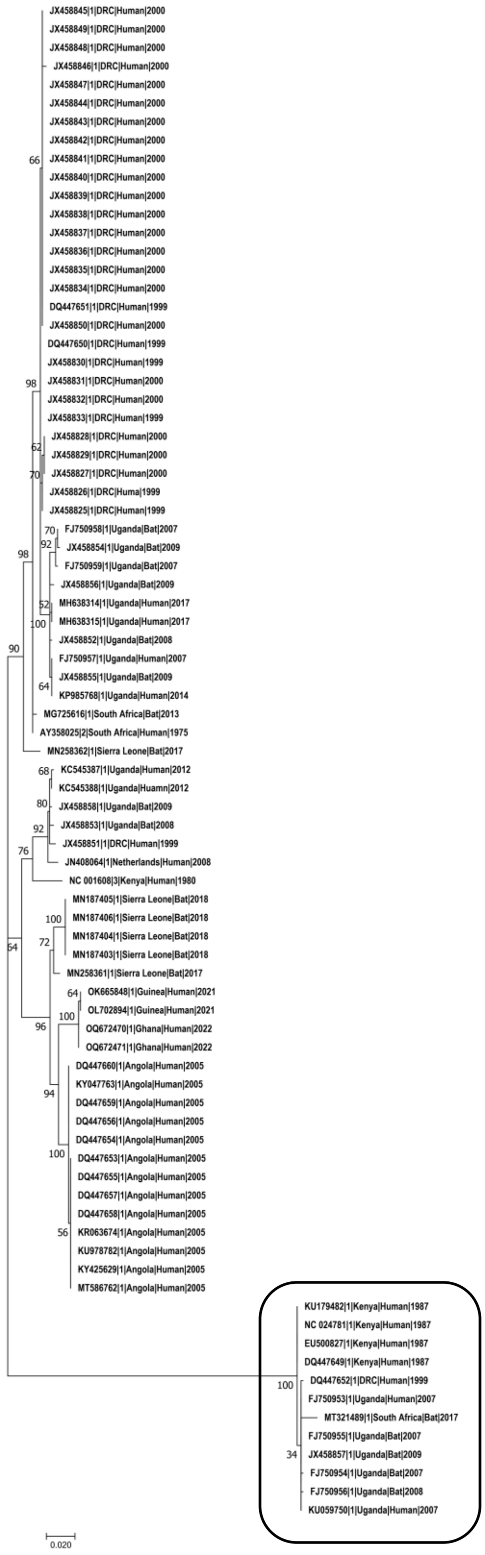

Figure S21: Maximum-Likelihood Phylogenetic Tree. The phylogenetic tree of MARV and RAVV L gene sequences alongside. The black box indicates RAVV sequences. Bootstrap values supporting the clustering of branches are indicated next to the corresponding branches.

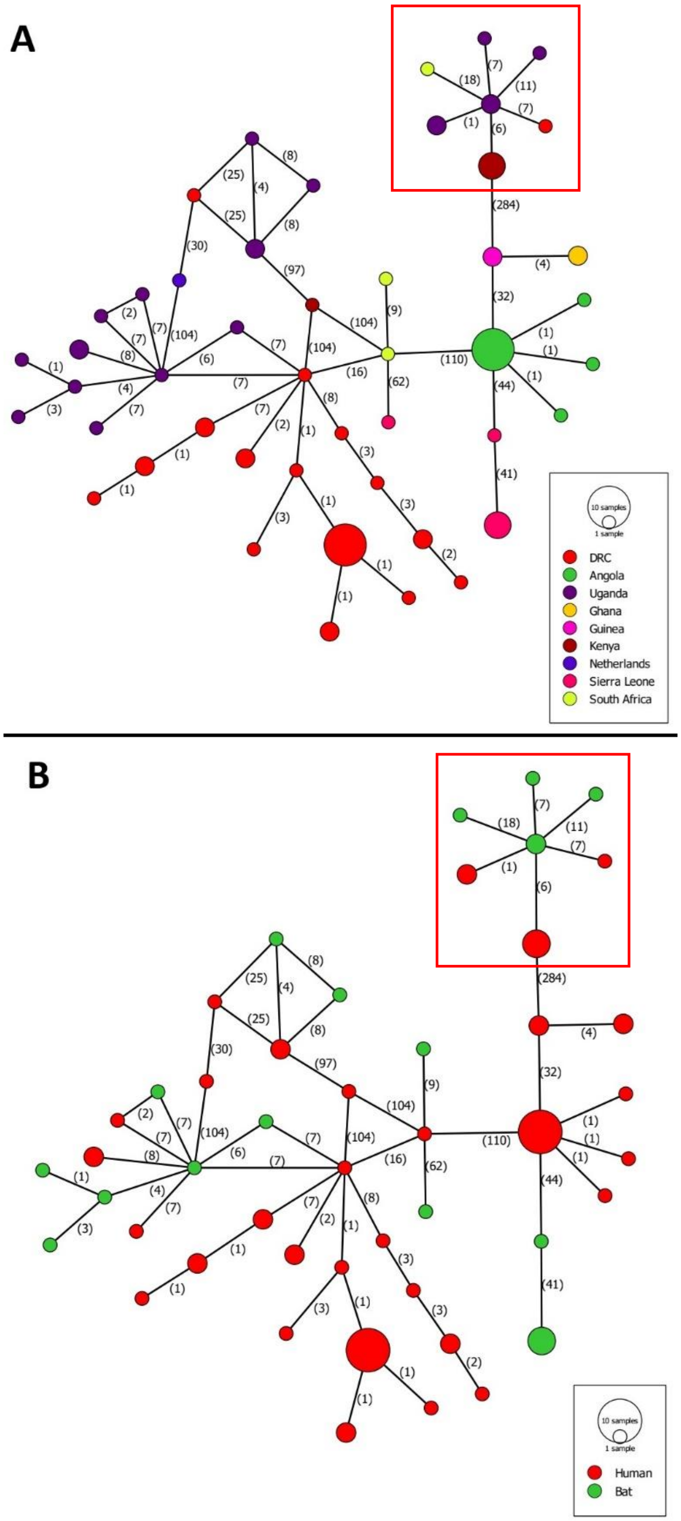

Figure S22: Minimum Spanning haplotypes networks of MARV and RAVV NP gene sequences. A: represent distribution of haplotypes according to countries of isolation. B: represent distribution of haplotypes according to host of isolation. The number of mutations between each haplotype is indicated between parentheses. Red lines highlight haplotypes related to RAVV sequences.

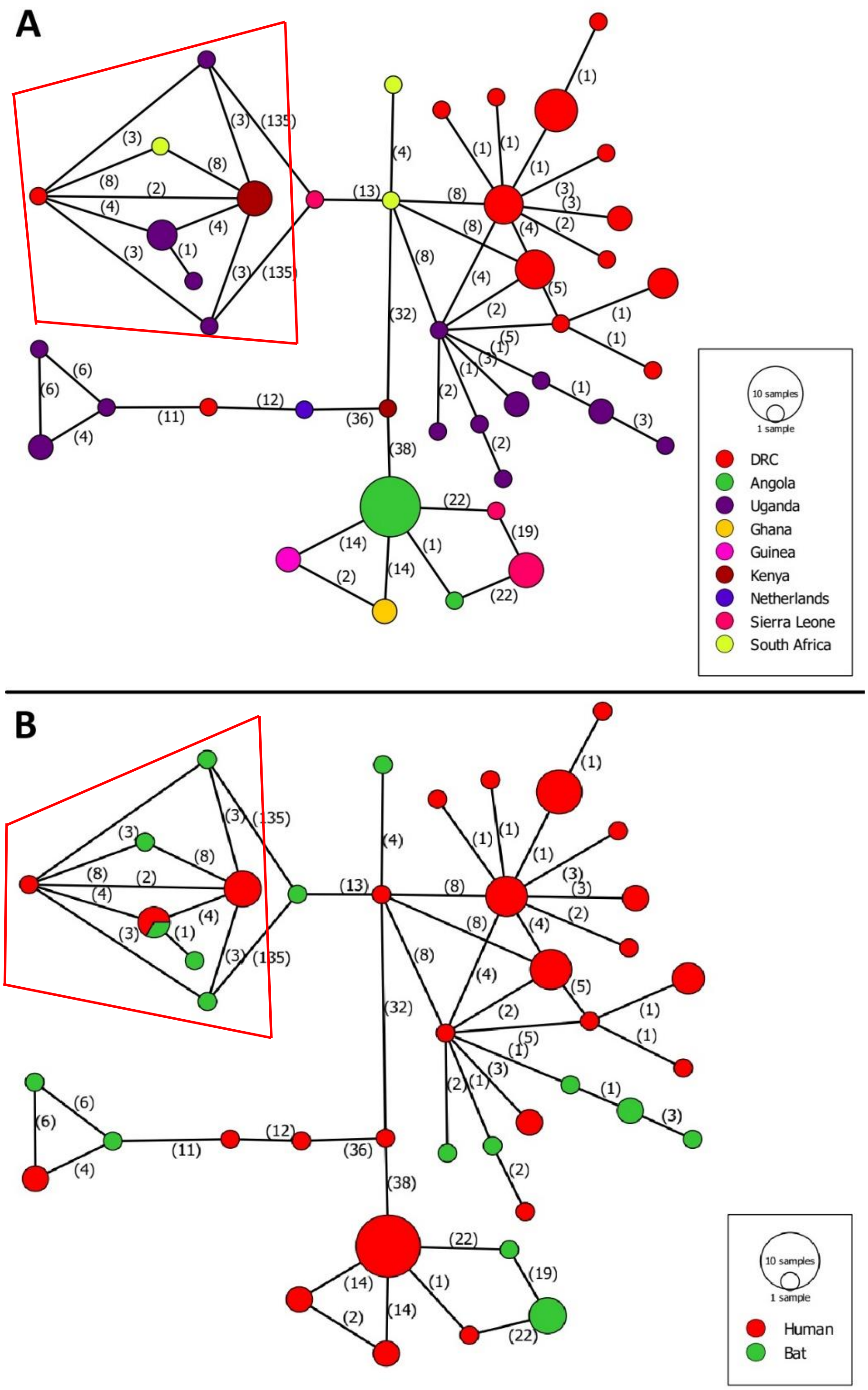

Figure S23: Minimum Spanning haplotypes networks of MARV and RAVV VP35 gene sequences. A: represent distribution of haplotypes according to countries of isolation. B: represent distribution of haplotypes according to host of isolation. The number of mutations between each haplotype is indicated between parentheses. Red lines highlight haplotypes related to RAVV sequences.

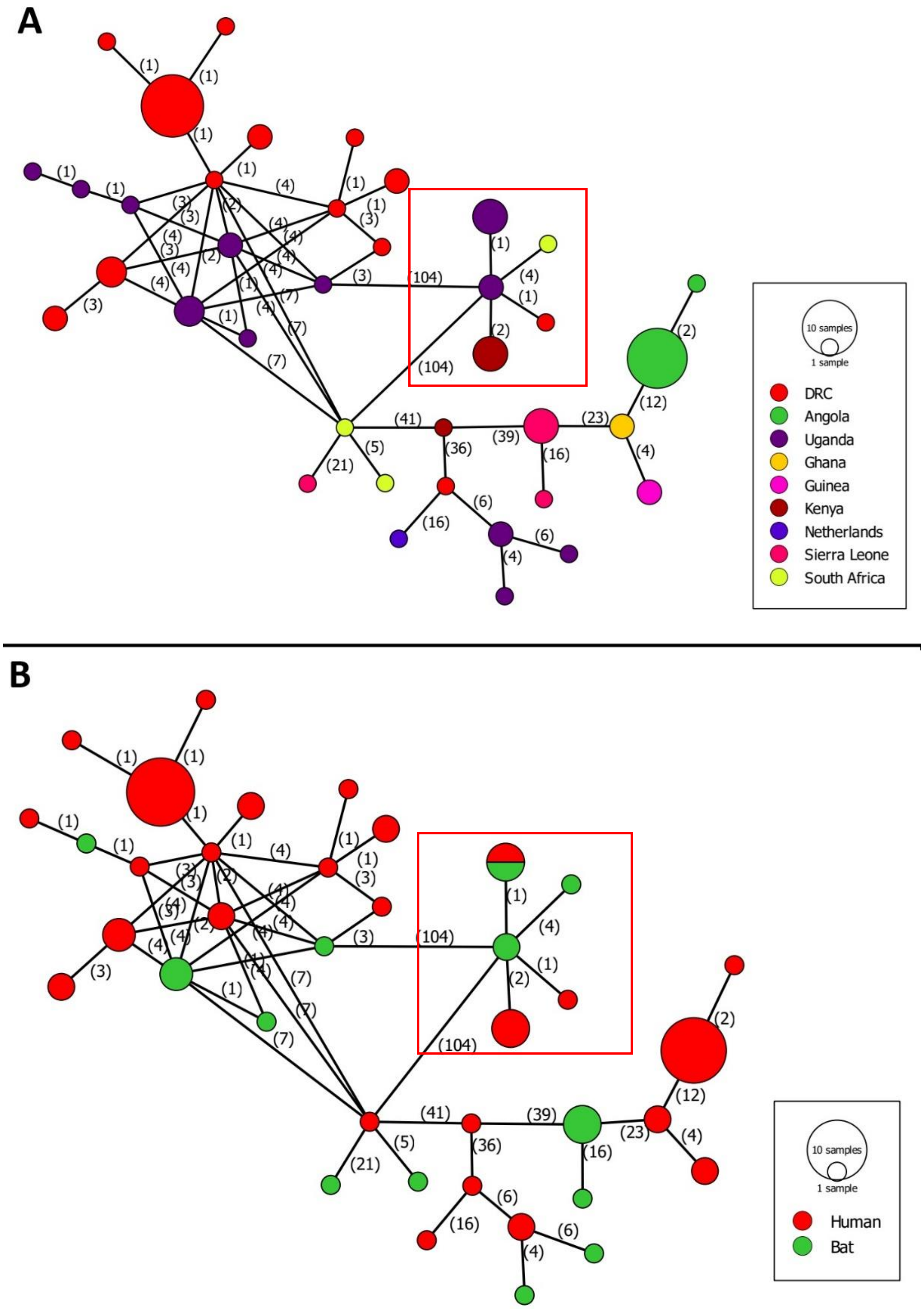

Figure S24: Minimum Spanning haplotypes networks of MARV and RAVV VP40 gene sequences. A: represent distribution of haplotypes according to countries of isolation. B: represent distribution of haplotypes according to host of isolation. The number of mutations between each haplotype is indicated between parentheses. Red lines highlight haplotypes related to RAVV sequences.

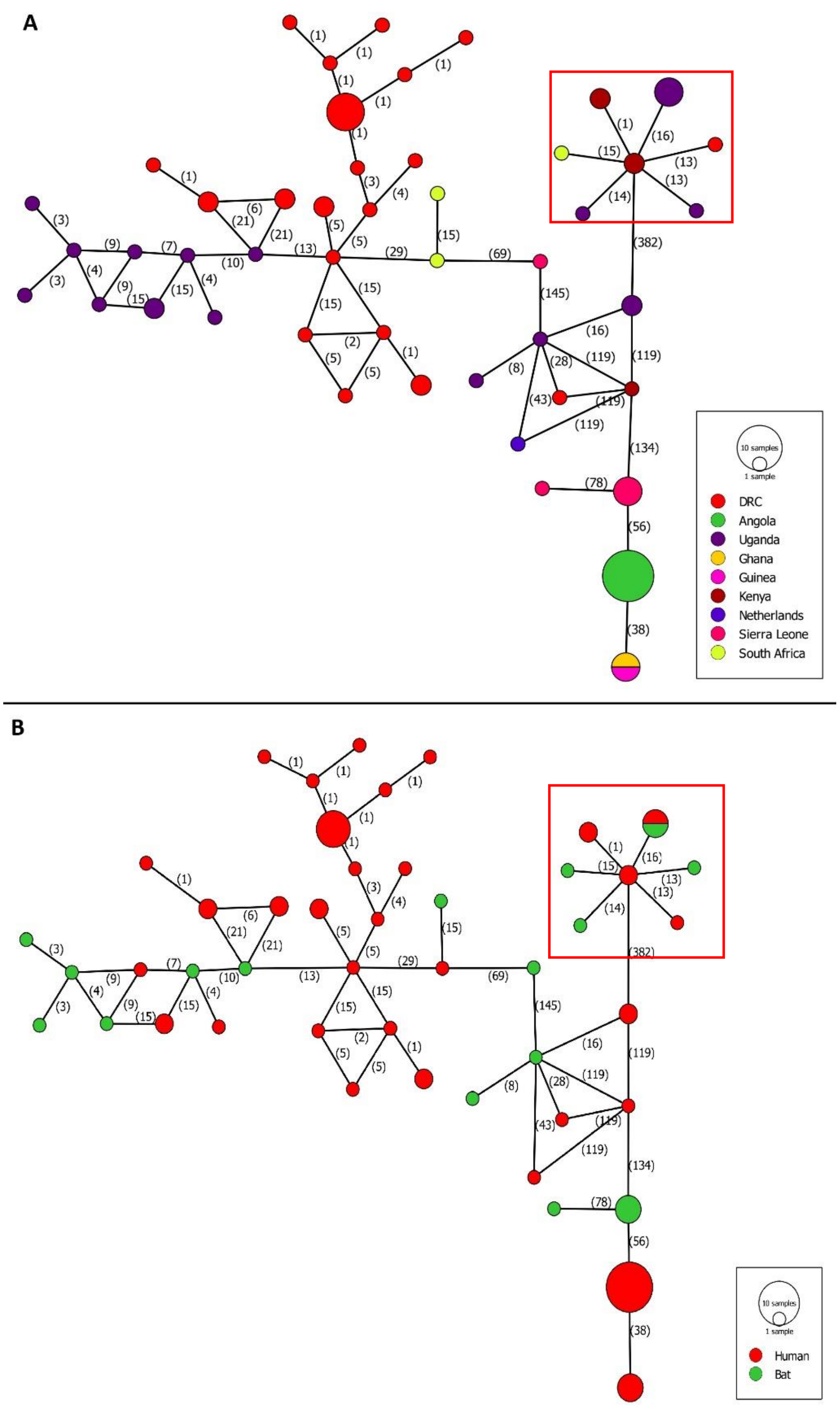

Figure S25: Minimum Spanning haplotypes networks of MARV and RAVV GP gene sequences. A: represent distribution of haplotypes according to countries of isolation. B: represent distribution of haplotypes according to host of isolation. The number of mutations between each haplotype is indicated between parentheses. Red lines represent highlight related to RAVV sequences.

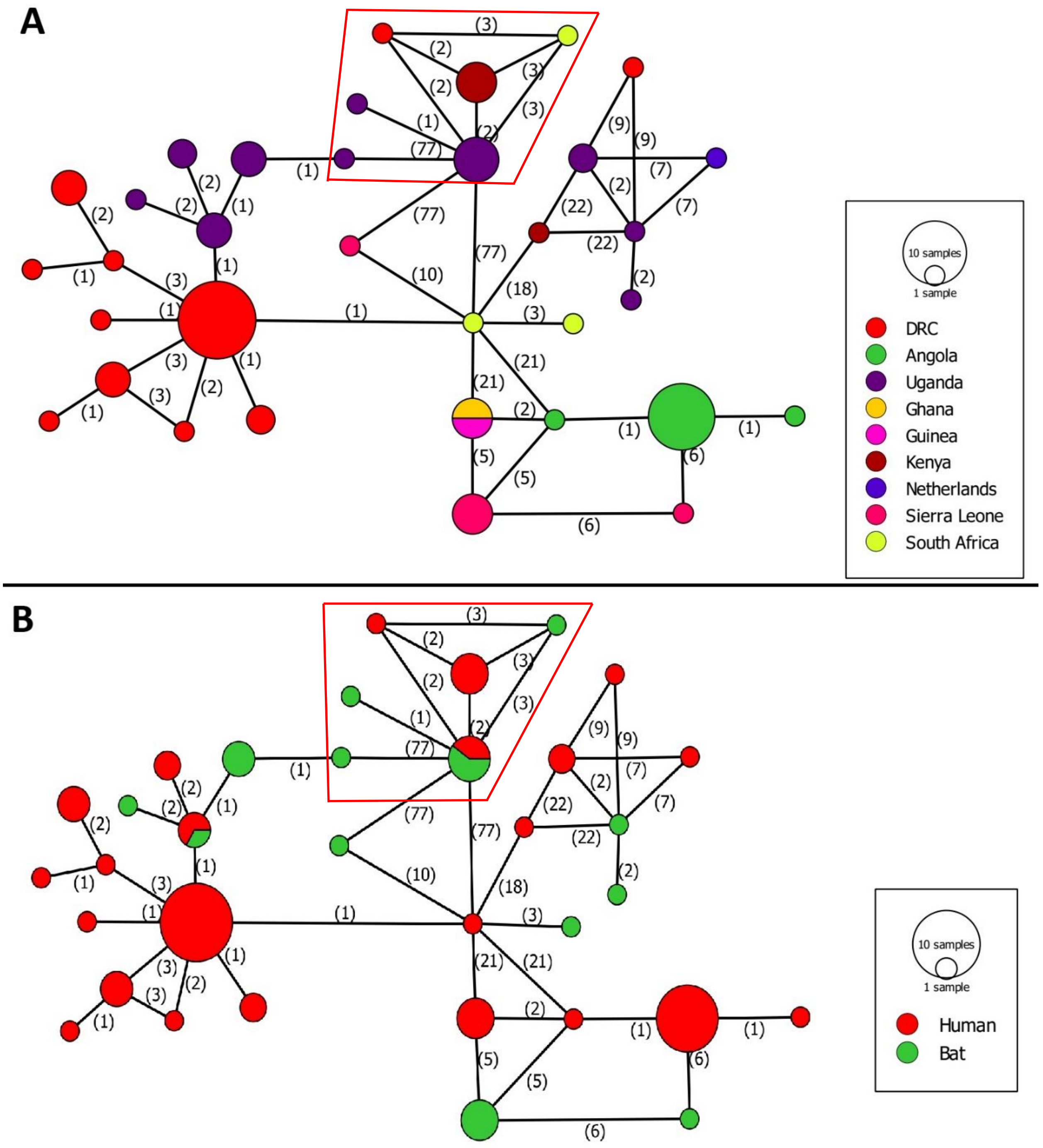

Figure S26: Minimum Spanning haplotypes networks of MARV and RAVV VP30 gene sequences. A: represent distribution of haplotypes according to countries of isolation. B: represent distribution of haplotypes according to host of isolation. The number of mutations between each haplotype is indicated between parentheses. Red lines highlight haplotypes related to RAVV sequences.

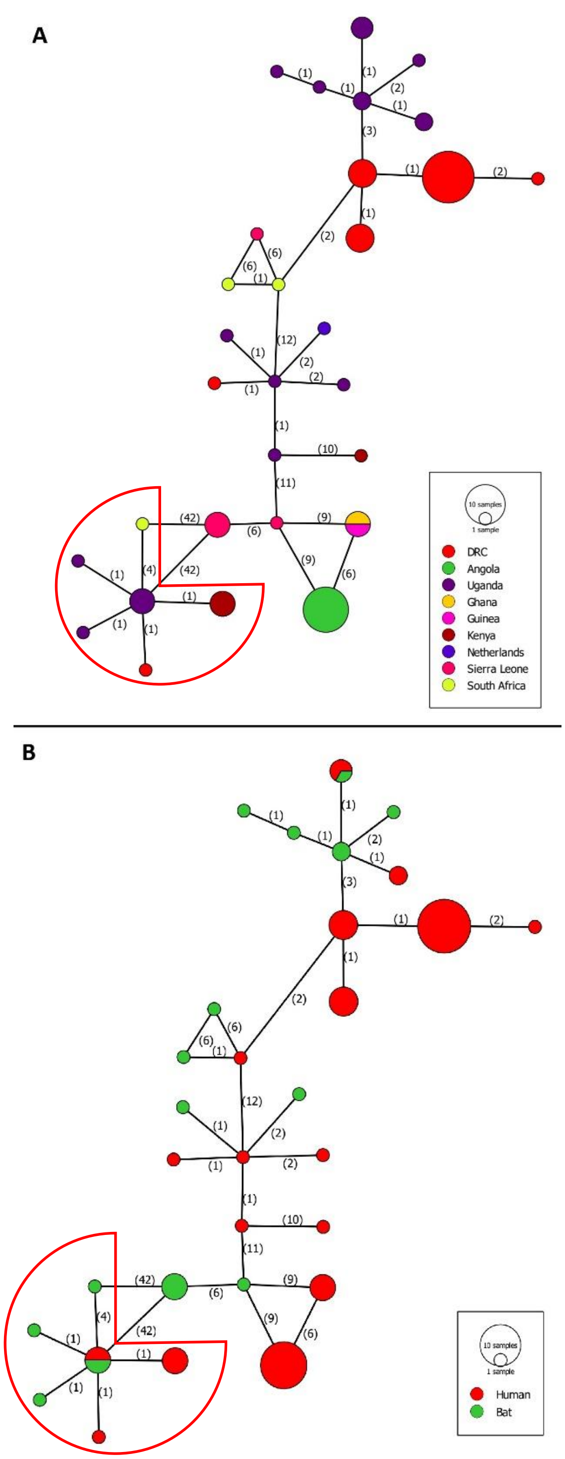

Figure S27: Minimum Spanning haplotypes networks of MARV and RAVV VP24 gene sequences. A: represent distribution of haplotypes according to countries of isolation. B: represent distribution of haplotypes according to host of isolation. The number of mutations between each haplotype is indicated between parentheses. Red lines highlight haplotypes related to RAVV sequences.

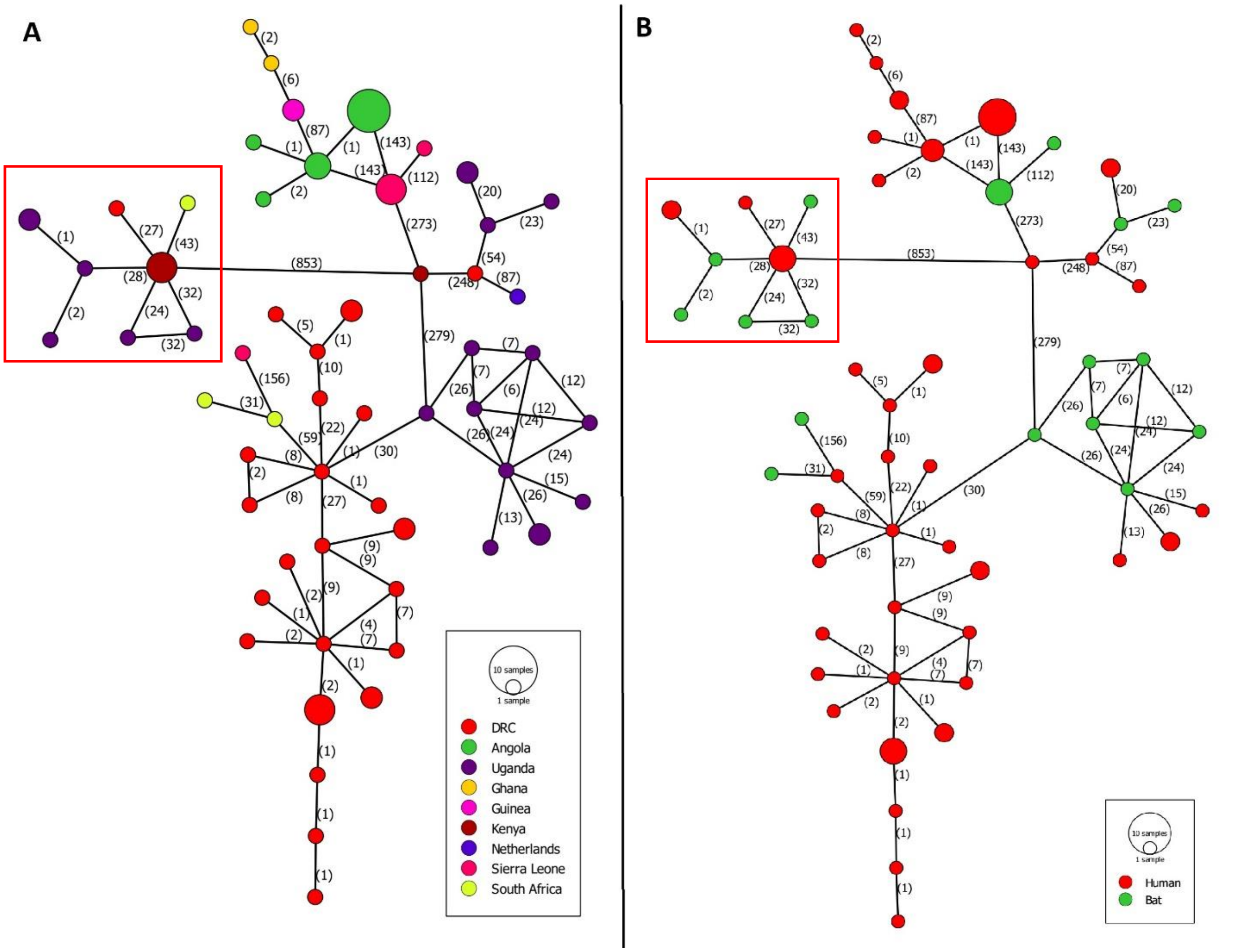

Figure S28: Minimum Spanning haplotypes networks of MARV and RAVV L gene sequences. A: represent distribution of haplotypes according to countries of isolation. B: represent distribution of haplotypes according to host of isolation. The number of mutations between each haplotype is indicated between parentheses. Red lines highlight haplotypes related to RAVV sequences.
